# Bidirectional Communication between the Pontine Nucleus Incertus and the Medial Septum Is Carried Out by Electrophysiologically-Distinct Neuronal Populations

**DOI:** 10.1523/JNEUROSCI.0230-21.2022

**Published:** 2022-03-16

**Authors:** Aleksandra Trenk, Magdalena Walczak, Agata Szlaga, Kamil Pradel, Anna Blasiak, Tomasz Blasiak

**Affiliations:** Department of Neurophysiology and Chronobiology, Institute of Zoology and Biomedical Research, Jagiellonian University, 30-387, Krakow, Poland

**Keywords:** delta waves, electrophysiology, medial septum, nucleus incertus, theta rhythm

## Abstract

Theta oscillations are key brain rhythm involved in memory formation, sensorimotor integration, and control of locomotion and behavioral states. Generation and spatiotemporal synchronization of theta oscillations rely on interactions between brain nuclei forming a large neural network, which includes pontine nucleus incertus (NI). Here we identified distinct populations of NI neurons, based on the relationship of their firing to hippocampal waves, with a special focus on theta oscillations, and the direction and type of interaction with the medial septum (MS) in male, urethane-anesthetized rats. By recording NI neuronal firing and hippocampal LFP, we described NI neurons that fire action potentials in a theta phase-independent or theta phase-locked and delta wave-independent or delta wave-locked manner. Among hippocampal activity-independent NI neurons, irregular, slow-firing, and regular, fast-firing cells were observed, while hippocampal oscillation-/wave-locked NI neurons were of a bursting or nonbursting type. By projection-specific optotagging, we revealed that only fast-firing theta phase-independent NI neurons innervate the MS, rarely receiving feedback information. In contrast, the majority of theta-bursting NI neurons were inhibited by MS stimulation, and this effect was mediated by direct GABAergic input. Described NI neuronal populations differ in reciprocal connections with the septohippocampal system, plausibly forming separate neuronal loops. Our results suggest that theta phase-independent NI neurons participate in theta rhythm generation through direct innervation of the MS, while theta-bursting NI neurons further transmit the rhythmic signal received from the MS to stabilize and/or strengthen rhythmic activity in other structures.

**SIGNIFICANCE STATEMENT** The generation and spatiotemporal synchronization of theta oscillations rely on interactions between nuclei forming a large neural network, part of which is the pontine nucleus incertus (NI). Here we describe that within NI there are populations of neurons that can be distinguished based on the relationship of their firing to hippocampal theta oscillations and delta waves. We show that these neuronal populations largely do not have reciprocal connections with the septohippocampal system, but form separate neuronal loops. Our results suggest that medial septum (MS)-projecting, fast-firing, theta phase-independent NI neurons may participate in theta rhythm generation through direct innervation of the MS, while theta-bursting NI neurons may further transmit the rhythmic signal received from the MS to other structures.

## Introduction

Theta rhythm is one of the most abundant brain oscillatory phenomena, characterized by frequency in the 3–12 Hz band. This rhythm can be observed at the level of the electrical activity of neuronal populations [e.g., local field potential (LFP); Bland, 1986], in the rhythmic pattern of action potential firing of multiple or single neurons (e.g., rhythmic theta bursts; Bland et al., 2002; Di Prisco et al., 2002), and in the oscillations of neuronal membrane potential (Nuñez et al., 1987; Strata, 1998; Bland et al., 2002). Theta rhythms present at various levels of the organization of the brain may interfere with each other and synchronize themselves in a large number of interconnected (directly and/or indirectly) structures (Buzsáki and Moser, 2013). Theta oscillations, spatiotemporally synchronized in nuclei-forming functional networks, are a source of timing information used for memory coding and retrieval, sensorimotor integration, and processing of spatial information (Bland, 1986; Pavlides, et al., 1988; Buzsáki, 1989; Huerta and Lisman, 1993; O'Keefe and Recce, 1993; Oddie and Bland, 1998; Buzsáki, 2002; Vertes, 2005).

Several lines of evidence have shown that the frequency and amplitude of hippocampal theta oscillations depend on the synergistic action of the bursting and tonic firing of theta-ON neurons located in the nuclei of the ascending brainstem hippocampal synchronizing pathway. Tonically firing theta-ON neurons predominates at brainstem levels of this system, while bursting theta-ON neurons are more often found at higher brain levels (Vertes et al., 2004). However, it has been shown that bursting theta-ON neurons can be found at the level of brainstem and midbrain nuclei, plausibly participating in the synchronization and functional coupling of widely distributed structures responsible for different mnemonic functions (Kocsis et al., 2001; Di Prisco et al., 2002; Ma et al., 2013).

An important element of the ascending brainstem hippocampal synchronizing pathway is the nucleus incertus (NI; Ryan et al., 2011; Brown and McKenna, 2015; Korotkova et al., 2018; Ma et al., 2018). In the rat, the NI is a group of large, mostly GABAergic neurons located below the IV ventricle, expressing several markers, including relaxin-3 (RLN3), neuromedin B (NMB) and the corticotropin-releasing factor receptor 1 (Banerjee et al., 2010; Ma et al., 2013; Lu et al., 2020). NI neurons strongly innervate elements of the septohippocampal system (Goto et al., 2001; Olucha-Bordonau et al., 2003; Teruel-Martí et al., 2008). In the rat medial septum (MS), afferents from NI form a dense plexus of symmetric (putatively inhibitory) terminals that synapse on the hippocampus-projecting neurons (Olucha-Bordonau et al., 2012). Activation or inactivation of NI has been shown to induce or attenuate hippocampal theta rhythm, respectively (Nuñez et al., 2006). Strong coherence between the NI local field potential and hippocampal theta has been described, as well as theta phase-locked firing of some NI neurons (Cervera-Ferri et al., 2011; Ma et al., 2013). Interestingly, Martínez-Bellver et al. (2017) identified a causal interaction between firing of NI neurons and the septohippocampal system. Recent studies have shown that there is considerable heterogeneity within the NI, at the functional, neurochemical, and connectivity levels. It was shown that the optogenetic activation of a subpopulation of NMB-expressing NI neurons in the mouse increased, whereas optogenetic inhibition of these cells decreased the power of hippocampal theta rhythm (Lu et al., 2020). In contrast—as shown by another group—optogenetic activation of GABAergic NI neurons, which also includes NMB-expressing cells, decreased hippocampal theta power (Szőnyi et al., 2019; Nasirova et al., 2020). These data reveal that the manipulation of different populations of NI neurons has different effects, both at the hippocampal theta and behavioral levels. However, the electrophysiological identity of the NI neuronal subpopulations, their connections with the septohippocampal network, and their relationship with hippocampal theta rhythm have not been well defined to date.

Therefore, the aim of our study was to identify NI neuronal populations that innervate and/or receive innervation from the rat MS and to determine the temporal relationship of their firing with the hippocampal theta oscillations and delta waves, in order to better define neuronal circuits involved in hippocampal synchronization.

## Materials and Methods

### Experimental design

#### Animals and ethical approval.

Experiments were performed on male Sprague Dawley rats at an age of 8–12 weeks and a weight of 300–400 g. The animals were bred at the Institute of Zoology and Biomedical Research Animal Facility at the Jagiellonian University in Krakow under standard conditions (12 h light/dark cycle, light on at 8:00 A.M.; temperature, 20 ± 2°C; humidity, 50 ± 10%) with food and water available *ad libitum*. The experiments were approved by the second Local Institutional Animal Care and Use Committee (Krakow, Poland) and conducted in accordance with the EU Directive 2010/63/EU on the protection of animals used for scientific purposes. All efforts were made to minimize pain and discomfort, as well as the number of animals used in the procedures.

#### Viral vectors injections.

Anesthesia was induced by injection of a ketamine (100 mg/kg, i.p.; Ketamina; Biowet) and xylazine (10 mg/kg, i.p.; Sedazin) mixture and maintained by injection of an additional 30% dose of ketamine during the procedure, as required. An anti-inflammatory drug was injected subcutaneously at the beginning of the surgery (0.4% in saline; body weight, 0.1 ml/100 g; Tolfedine, Vetoquinol). After the disappearance of the corneal reflex and withdrawal response to paw pinch, the animal was mounted on the stereotaxic frame (model SF-1450AP, ASI Instruments) on standard ear and incisor bars. During the whole procedure, the body temperature of the animal was maintained at 37 ± 0.5°C by an automatic heating pad (model TCP-02, WMT). The eyes were protected from drying by the application of eye drops (Starazolin HydroBalance, Polpharma). After cutting the skin and exposing the bones of the skull, a trepanation hole was drilled to allow stereotactic access to the MS. A glass microcapillary tube (VITREX) was processed on a vertical puller (model PE-21, Narishige) to obtain an ultrathin tip that was then broken to the final diameter of 40–50 μm. The injection needle prepared in this way was tightly connected to a syringe (1 μl; Hamilton), and the whole system was filled with paraffin oil (Sigma-Aldrich). The tip of the microinjection capillary was backfilled with the viral vector just before it was lowered into the target structure. The MS of 17 rats (*in vivo* recordings, 8 rats; *ex vivo* recordings, 9 rats; see below) was injected with an adeno-associated virus (AAV) vector carrying genes for channelrhodopsin-2 (ChR2) and enhanced yellow fluorescent protein (EYFP), both expressed under the control of human synapsin (hSyn) promoter [AAV2-hSyn-hChR2(H134R)-EYFP; donated by Karl Deisseroth, UNC Vector Core, Chapel Hill, NC; Zhang et al., 2006]. The MS of 15 rats was injected with a retrograde adeno-associated viral vector carrying genes for Chronos and green fluorescent protein (GFP), both expressed under the control of the synapsin promoter (pAAV-Syn-Chronos-GFP; donated by Edward Boyden (McGovern Institute for Brain Research, MIT, Cambridge, MA); viral preparation #59170-AAVrg, Addgene; Klapoetke et al., 2014). The following coordinates were used for MS injections: anteroposterior (AP), +0.6 mm; mediolateral (ML), 0 mm; dorsoventral (DV), −7.2 mm (first injection) and −6.6 mm (second injection) ventral from bregma (300 nl was injected at each ventral point). Finally, the scalp was sutured, and the wound was covered with spray dressing (Nanosilver, Bioton) to promote healing. After surgery, the animals were allowed to recover for 2 weeks; and their condition was monitored daily until the final experiment.

#### *In vivo* preparation.

All *in vivo* electrophysiological experiments were performed on urethane anesthetized rat preparations (urethane injection; 1.4 g/kg, i.p., diluted in saline; Sigma-Aldrich). The anesthetized animal was mounted in the stereotaxic frame (model SF-1450AP, ASI Instruments) on standard ear and incisor bars. A tracheotomy was performed to reduce the mechanical instability of the brainstem resulting from the respiratory movements of the animal. During the whole experiment, the electrocardiogram was monitored and body temperature was held at 37 ± 0.5°C using an automatic heating pad (model TCP-02, WMT). After cutting the skin and exposing the bones of the skull, the bregma point was set below the level of the λ point (usually, 2.3 mm) to obtain a rostral inclination of the skull of 15°, which allowed access to the NI bypassing the confluence of the superior sagittal sinus and transverse sinuses. Craniotomies were made above the areas of insertion of the electrodes and optical fibers, and, after cleaning, the exposed brain surface was protected with a drop of paraffin oil (Sigma-Aldrich). The stereotactic coordinates of the examined brain structures were taken from the rat brain atlas (Paxinos and Watson, 2007) and then recalculated taking into account the rostral inclination of the skull.

#### Recording of the hippocampal local field potential.

LFPs were recorded with a blunt-cut, Teflon-coated stainless steel wire (outer diameter, 200 µm; Science Products) from stratum lacunosum-moleculare (SLM) of the hippocampal CA1 field (rostral angle, 15°; AP, −4.6 mm; ML, 3 mm; DV, −1.9 mm from bregma). To verify the placement of the electrode, an electrolytic lesion [current, 0.6 mA (applied for 1 s)] was made at the end of the recording. In the case of experiments with optogenetics, the LFP recording electrode was attached to the skull with dental cement (Duracryl Plus, SpofaDental).

#### Multichannel *in vivo* recordings from the NI.

The 32-channel multielectrode array (MEA; four shanks, eight electrodes per shank; model A4x8-10 mm-50–200-177, NeuroNexus) was positioned in the NI using the following stereotaxic coordinates: AP, −9.2 to −9.7 mm; ML, 0.1–0.4 mm; DV, −7.5 to −7.2 mm from bregma (Paxinos and Watson, 2007). The spontaneous firing activity of NI neurons was recorded in one position in each animal. The extracellular signal picked up by MEA was digitized (40 kHz/channel), wide-band filtered (0.77–7500 Hz), and stored on a hard drive using OmniPlex D Neural Data Acquisition System (Plexon). To reconstruct the position of each MEA, its shanks were covered with a fluorescent dye (DiI; Thermo Fisher Scientific) before brain implantation.

#### Optogenetic stimulation of the MS during the multichannel recording of NI electrical activity *in vivo*.

Three weeks after the viral vector injection into the MS (AAV2-hSyn-hChR2(H134R)-EYFP; as described above), responses of NI neurons to MS optogenetic stimulation were observed. The LFP signal was recorded from the stratum lacunosum-moleculare of the hippocampal CA1 field, and electrical activity of NI neurons was extracellularly recorded with a multielectrode array. For optogenetic stimulation, the optical fiber (core ∅ = 100 µm; numerical aperture (NA), 0.22; Thorlabs) was implanted so that its tip was aimed at the MS (coordinates of the tip: right angle, 10°; AP, 0.7 mm; ML, 0.7 mm; DV, −5.6 mm from the bregma; Paxinos and Watson, 2007). The MEA and optical fiber, were both covered with a fluorescent dye (DiI; Thermo Fisher Scientific) before brain implantation to help verify their placement. The optical fiber was connected to a blue laser light source (wavelength, 473 nm; model MBL-III-473 with PSU-III-LED controller, CNI Optoelectronics Technology Co., Ltd.). The light power never exceeded 10 mW, as measured with the optical power meter (model PM-100D equipped with S121C photodiode, ThorLabs) at the tip of the optical fiber. Temporal parameters of the light pulses were digitally controlled by a micro1401 mk II laboratory interface and Spike2 software running a custom-written script (Cambridge Electronic Design). Protocols of optogenetic stimulation of MS included the following three frequencies from the theta band: 4 Hz (4 × 100 ms light pulses), 8 Hz (8 × 40 ms light pulses), 12 Hz (12 × 20 ms light pulses), one high-frequency (20 Hz) pattern (40 × 10 ms light pulse), and one single-pulse (50 ms) pattern. The given frequencies are based on intervals between the onsets of light pulses. Stimulation at each frequency/pattern was applied at least 50 times at 10 s intervals.

#### *Ex vivo* recording: tissue preparation, data acquisition, and optogenetic stimulation of MS originating terminals.

Whole-cell patch-clamp electrophysiological recordings were performed as previously described (Kania et al., 2020). Male Sprague Dawley rats were deeply anesthetized with isoflurane (Aerrane, Baxter) and decapitated between zeitgeber time 2 (ZT2) and ZT3. Brains were collected in ice-cold, low-sodium, high-magnesium ACSF, containing the following (in mm): 185 sucrose, 25 NaHCO_3_, 3 KCl, 1.2 NaH_2_PO_4_, 2 CaCl_2_, 10 MgSO_4_, and 10 glucose, pH 7.4, with osmolality of 290–300 mOsmol/kg, and cut into 250-μm-thick coronal sections on a vibrating microtome (model VT1000S; Leica Instruments). Sections containing the NI were transferred to an incubation chamber containing carbogenated and warm (32°C) ACSF, and containing the following (in mm): 118 NaCl, 25 NaHCO_3_, 3 KCl, 1.2 NaH_2_PO_4_, 2 CaCl_2_, 1.3 MgSO_4_, and 10 glucose, pH 7.4, with osmolality of 290–300 mOsmol/kg. After a recovery period (90–120 min), the slices were placed in a recording chamber, where the tissue was perfused (2 ml/min) with carbogenated, warm (32°C) ACSF of the same composition as in the incubation chamber. MS was also cut into 250-μm-thick coronal sections on a vibrating microtome, and the viral vector injection sites were verified using fluorescent microscope (Imager M2, Axio) with an A-Plan 10×/0.25 objective. Recording micropipettes (7–9 MΩ; measured in the recording chamber filled with ACSF) were fabricated from borosilicate glass capillaries (model BF100-78–10, Sutter Instruments) using a horizontal puller (model P-97, Sutter Instruments) and filled with an intrapipette solution containing the following (in mm): 145 potassium gluconate, 2 MgCl_2_, 4 Na_2_ATP, 0.4 Na_3_GTP, 5 EGTA, and 10 HEPES, pH 7.3, with osmolality of 290–300 mOsmol/kg, and biocytin (0.05%, for subsequent immunofluorescent identification of recorded neurons). The calculated liquid junction potential was +15 mV, and this value was subtracted from the recorded signal before the analysis. NI neurons were approached with the tip of the recording micropipette under visual control using an upright microscope (Examiner D1, Zeiss) equipped with video-enhanced infrared differential interference contrast. Cell-attached and subsequent whole-cell configurations were obtained using a negative pressure delivered by mouth suction. Voltage-clamp recording and data acquisition were performed using an SEC 05LX amplifier (NPI) and a Micro1401 mk II converter (Cambridge Electronic Design) operated by Signal and Spike2 software (Cambridge Electronic Design). Analogue signal was filtered (low-pass filter, 3 kHz) and digitized (20 kHz). All drugs used in the *ex vivo* experiments were applied via a perfusion system. Voltage-clamp recordings (command potential, −50 mV) were performed in standard ACSF or ACSF containing gabazine (5 μm; catalog #1262, Tocris Bioscience), a selective GABA_A_ receptor antagonist. As the calculated reversal potential for Cl^–^ currents for the solutions used in this study was −90 mV, outward currents recorded at –50 mV holding potential were considered inhibitory (IPSCs), while inward currents were considered excitatory (EPSCs). The tip of the optical fiber (core ∅ = 200 µm; NA, 0.5; Thorlabs) was positioned ∼100 µm above the surface of the brain slice, aiming at the tip of the recording micropipette positioned within the NI. The optical fiber was connected to a blue light-emitting LED (wavelength, 465 nm; PlexBright LED Module, Plexon). The power of the LED output was controlled by the current source (PlexBright LED Driver LD-1, Plexon) and never exceeded 10 mW, as measured with an optical power meter (model PM-100D equipped with a model S121C photodiode, ThorLabs) at the tip of the optical fiber. The temporal parameters of the light pulses were digitally controlled by a micro1401 mk II laboratory interface and Spike2 software running a custom-written script. Optogenetic stimulation of MS originating terminals within the NI consisted of at least 20× 5 ms light pulses applied at 10 s intervals. After the experiment, brain slices were fixed and processed for histologic examination as described below.

#### Optogenetic identification of NI neurons innervating the MS.

Three weeks after the retrograde viral vector injection into the MS (pAAV-hSYN-Chronos-GFP; as described above), the NI neurons innervating the MS were optogenetically identified. The LFP signal was recorded from the hippocampal CA1 field, and the electrical activity of NI neurons was extracellularly recorded with the multielectrode array, as described above. The optical fiber (core ∅ = 200 µm; NA, 0.5; Thorlabs) was implanted above the NI (coordinates of the tip: caudal angle, 30°; AP, −9.6 mm; ML, 0 mm; DV, −7.2 mm from bregma; Paxinos and Watson, 2007). Before implantation, the MEA and the optical fiber were covered with a fluorescent dye (DiI and DiD, respectively; Thermo Fisher Scientific) to allow for the subsequent verification of their location in the tissue. The optical fiber was connected to a blue light-emitting LED (wavelength, 465 nm; model PlexBright LED Module, Plexon). The power of the LED output was controlled by the precise current source (model PlexBright LED Driver LD-1, Plexon) and never exceeded 12 mW, as measured with an optical power meter (model PM-100D equipped with a S121C photodiode, ThorLabs) at the tip of the optical fiber. Protocols of optogenetic stimulation of NI included eight patterns, each consisting of 50 light pulses, as follows: 5 ms pulses applied at frequencies of 10, 30, and 50 Hz, and 50 ms pulses applied at 1 Hz. A neuron was considered to be directly driven by optogenetic stimulation (i.e., optogenetically tagged) if it responded with 100% fidelity to 10 and 30 Hz pulses, with at least 90% fidelity to 50 Hz pulses, and with high-frequency discharges (>50 Hz) to laser light pulses of 50 ms duration.

#### Histologic verification, immunohistochemical staining, and image acquisition.

At the end of each *in vivo* experiment, rats were transcardially perfused with PBS followed by 4% formaldehyde solution. The extracted brains (*in vivo* experiments) or brain slices (*ex vivo* experiments) were kept overnight in 4% formaldehyde solution at 4°C. Brains were cut into 50 µm coronal slices on a vibrating microtome (model VT1000S, Leica) and the brain slices from the *ex vivo* experiments were processed histologically without further sectioning. To visualize biocytin-filled neurons recorded *ex vivo*, fixed free-floating sections were blocked and permeabilized with 10% normal donkey serum (NDS) and 0.6% Triton X-100 in PBS, respectively, at 4°C overnight or for 3 h at room temperature. Subsequently, after washing in PBS, sections were incubated with ExtrAvidin-Cy3 (1:200), 2% NDS, and 0.3% Triton X-100 in PBS for 48 h and up to 72 h at 4°C, and, after several washing steps (in PBS), the slices were mounted on glass slides and coverslipped with Fluoroshield with DAPI (Sigma-Aldrich). Brain sections from the *in vivo* experiments involving pAAV-Syn-Chronos-GFP injection in to the MS (see above) were immunostained against GFP to improve the visibility of cells transfected with the viral vector. Sections were placed in 10% NDS (Abcam, UK) and 0.6% Triton X-100 (Sigma-Aldrich) in PBS for 1 h at room temperature to block nonspecific binding sites and permeabilization, respectively. After washing in PBS, the slices were incubated for 24 h with rabbit anti-GFP antibodies (1:1000; Abcam). Then, after another washing step, the sections were incubated overnight in the secondary donkey anti-rabbit Alexa Fluor 488 antibody solution (1:400; Jackson ImmunoResearch). After final rinsing, the sections were placed on slides and coverslipped using Fluoroshield with DAPI (Sigma-Aldrich). Sections were imaged with a fluorescent microscope (Axio Imager.M2, AxioCam MRm camera, and A-Plan 10×/0.25 and EC-Plan-Neofluar 20×/0.25 objectives, Zeiss).

### Data processing and analysis

All data were processed and analyzed offline, except for the initial filtration of recorded signals as described. The extracellular signal from the MEA recording spots located within the NI (as demonstrated by histologic verification) was digitally filtered (high-pass filter, 300 Hz) and subjected to spike detection in Kilosort2 software (Pachitariu et al., 2016). The filtered signal and spike times of the sorted units were converted to SON file format using a custom-written MATLAB script (MathWorks), and then the quality of spike sorting was inspected and verified manually in Spike2 software (Cambridge Electronic Design). The units that had percentage of refractory period violations [(interspike intervals < 2 ms/total number of intervals) *100] >0.1% were rejected from further analysis. The final analysis of electrophysiological signals was conducted in NeuroExplorer and MATLAB software (NexTechnologies). Theta and delta epochs were identified based on the percentage of spectral power exceeding 50% in the theta band (3–6 Hz) and delta band (0.1–3 Hz), respectively, calculated for consecutive 4 s windows of the LFP signal recorded from the CA1 field (as described). The full band (0–500 Hz) power spectral density was calculated with fast Fourier transform applied to the LFP signal with Welch's method (4 s windows). Zero phases (i.e., peak negativity) of theta and delta waves were detected with NeuroExplorer software algorithm (NexTechnologies) based on the method described previously (Klausberger et al., 2003). Using a custom MATLAB script, each spike generated by the NI neuron was assigned a theta oscillation phase that was determined using the Hilbert transform. Based on the obtained values, the activity of each neuron was subjected to circular statistics using the MATALB CircStat toolbox (Berens, 2009) to determine whether, and if so, at which phase of hippocampal theta oscillation the given neuron preferentially generates action potentials. It was assumed that the firing of a neuron is theta phase independent if the probability of generating an action potential was uniformly distributed around the hippocampal theta cycle (*p*_R_ > 0.05, Rayleigh's test for uniformity) or direction vector length (*r* < 0.2, at *p*_R_ ≤ 0.05). If the probability was nonuniformly distributed around the hippocampal theta cycle (*p*_R_ ≤ 0.05) and the length of the direction vector was ≥0.2, the neuron was identified as theta phase locked (Lasztóczi et al., 2011). The theta-phase preference of specific NI neuron populations was determined and compared using the Watson–Williams test (Watson and Williams, 1956). To determine whether the neuronal activity was locked with delta waves the peri-delta-wave zero-phase *z*-score histograms (10 ms bins) were generated using NeuroExplorer. The obtained histograms were analyzed using a custom-made MATLAB script. A fifth degree polynomial was fitted to each histogram and the times and amplitudes of minimum and maximum closest to zero-phase were extracted. When at least one peak exceeded a *z*-score value of 2.575 (99% confidence level), the neuronal activity was considered correlated with the delta wave.

For each NI neuron, the median firing rate in theta and delta epochs was determined. The firing pattern of the recorded neuron (regular, irregular, bursting, and nonbursting) was determined based on interspike interval and autocorrelation histograms generated for theta epochs. Bursts of action potentials and the parameters describing them were detected using a custom MATLAB script based on MLIB toolbox (www.mathworks.com/matlabcentral/fileexchange/37339-mlib-toolbox-for-analyzing-spike-data). For each neuron, in each subsequent theta epoch, the spike density function (SDF; Gaussian kernel, width, 25 ms) and its mean value were calculated. The beginning of the burst was set on the first spike after the SDF exceeded the mean value, and the end of the burst was set on the last spike before the SDF returned below the mean value. The following parameters describing theta bursts and intensity of bursting were determined for each NI bursting neuron: length of the burst, number of spikes in the burst, intraburst frequency, bursting rate (i.e., bursts fired in time unit), percentage of spikes generated in the bursts [i.e., (number of spikes in bursts/total number of spikes) * 100%], interburst interval, and coefficient of variation of the interburst intervals.

To determine whether neuron firing was correlated with each other, cross-correlograms (10 ms bin; delay, ±0.25 s) of activity in pairs of simultaneously recorded neurons were generated using NeuroExplorer. The obtained cross-correlograms were analyzed using a custom-made MATLAB script. A seventh-degree polynomial was fitted to each cross-correlogram, and the time and amplitude of the maximum closest to the zero lag were extracted, as well as the minimum preceding it. When at least one peak exceeded a *z*-score value of 2.575 (99% confidence level), the neuronal activity of the pair was considered correlated with one another.

To check whether the optogenetic stimulation of the MS elicited reactions of NI neurons *in vivo*, the peristimulus spike density function (Gaussian kernel, width = 50 ms) histograms were prepared using custom-made Spike2 software (Cambridge Electronic Design). Then, using custom-made MATLAB scripts, the signal was divided into episodes of activity either above or below the mean firing rate in the baseline, and, next, their lengths and mean firing rates were extracted. Following a multivariate Gaussian distribution was fitted to both parameters of baseline episodes, and anomaly detection for the postbaseline episodes was performed. As a criterion for the detection, the Mahalanobis distance was used; its value was set to 5.9915, which is equivalent to the χ^2^ distribution-derived probability of 0.05 for two variables. The episodes lying outside of the distribution latencies that were <50 ms from the stimulation onset were classified as effects; the adjacent significant episodes were merged.

The amplitude of the light-evoked postsynaptic currents recorded during *ex vivo* experiments was determined based on the average response to 20 consecutive pulses (Spike2 software, Cambridge Electronic Design). The distribution of the obtained parameters was tested for normality using the Shapiro–Wilk test. All values, because of their non-normal distribution, were presented as medians and ranged between the first and third quartiles. Statistical evaluation of the differences between the medians was made using the Wilcoxon test (for paired data) or the Mann–Whitney test (for unpaired data) implemented in GraphPad Prism software (version 6; GraphPad Software). Differences with *p* < 0.05 were considered statistically significant.

## Results

### Electrophysiological characterization of NI neurons *in vivo*

#### Firing rate of NI neurons during cortical activation and SWA

**Figure 1.**
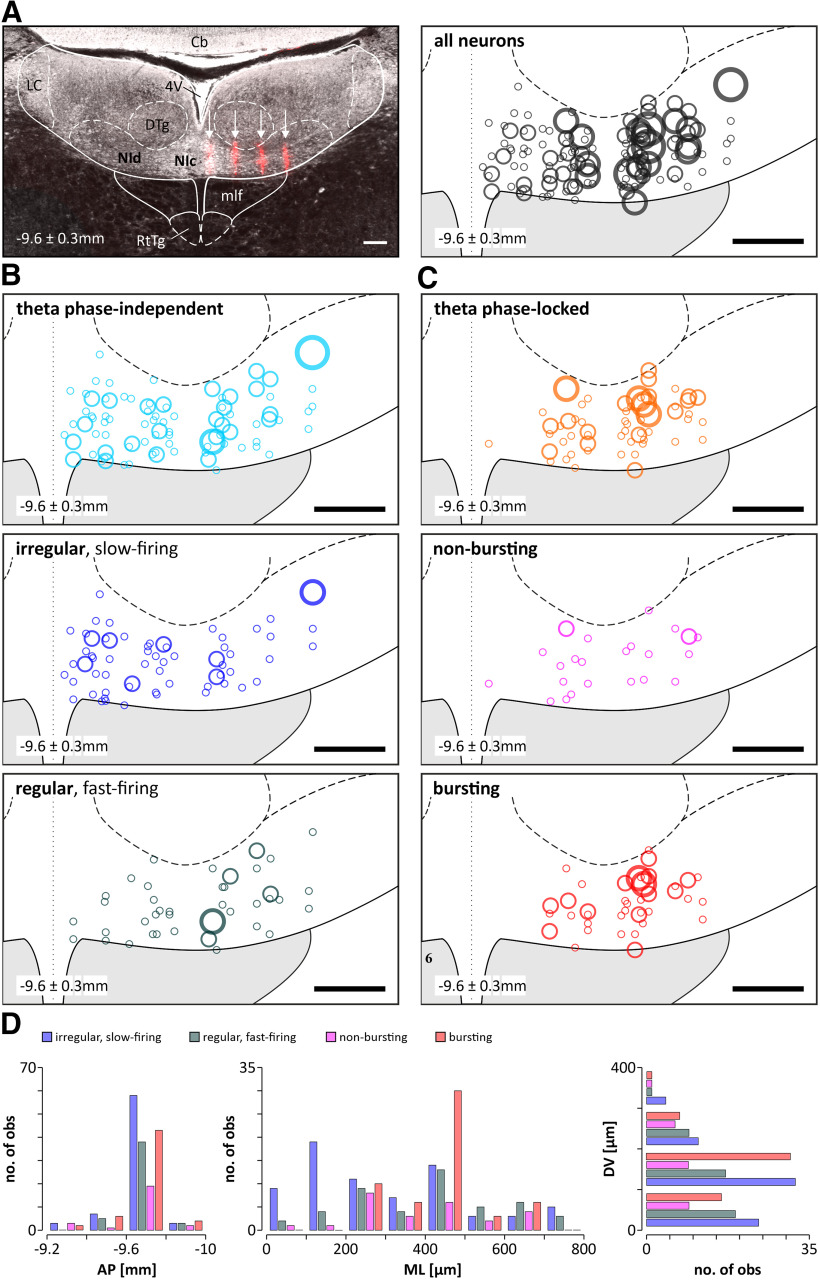
Histologic verification of the positioning of the recording MEA in the NI and reconstruction of the location of different types of neurons recorded within the NI.7 ***A***, Left, An example of the DiI-filled traces (red) of the four shanks of the MEA (indicated by the white arrows) terminating on the ventral border of the NI (the section is in the coronal plane and the MEA penetration was at a caudal angle of 15°; see Materials and Methods). Right, Reconstruction of the location of all recorded NI neurons (*n* = 197). ***B***, Reconstruction of the location of theta phase-independent neurons (top; *n* = 117) divided into irregular, slow-firing (middle; *n* = 71) and regular, fast-firing (bottom; *n* = 46) neurons. ***C***, location of theta phase-locked (top; *n* = 80) neurons in the NI divided into bursting (middle; *n* = 55) and nonbursting (bottom; *n* = 25) neurons. The size of the circles indicates the number of neurons recorded at a given location (○, ○, ○, ○ – 1, 2, 3, 4 neurons respectively). The dotted line indicates the midline. The location of the NI neurons is presented on one side, but the recordings were made from both sides of the brain. ***D***, Histogram ilustrating the distribution of different types of NI neurons along AP, ML, and DV axes (bin = 100 µm). Scale bars, 200 µm. Nic, NI pars compacta; NId, NI pars dissipata; 4V, fourth ventricle; Cb, cerebellum; DTg, dorsal tegmental nucleus; LC, locus coeruleus; mlf, medial longitudinal fasciculus; RtTg, reticulotegmental nucleus of the pons.

The position of the microelectrode array in the NI was confirmed in 16 animals (Fig. 1*A*). The activity of 197 NI neurons (average, 12 neurons/animal; range, 5–28 neurons/animal) was recorded over at least one full, spontaneous cycle of brain state alternation, covering the phase of cortical activation and slow-wave activity (SWA). The firing rate of the majority of NI neurons (87%, 171 of 197) was significantly higher during spontaneous cortical activation than during the SWA state (theta-ON neurons; activation: median, 19.6 Hz; first and third quartiles, 8.7 and 38.7 Hz; SWA: median, 9.8 Hz; first and third quartiles, 4.0 and 21.2 Hz; *n* = 171, *z* = 11.34, *p* < 0.0001, Wilcoxon matched-pairs test). The firing rate of the minority of NI neurons (3%, 6 of 197) did not change across brain states (theta unrelated; activation: median, 5.4 Hz; first and third quartiles, 3.9 and 26.9 Hz; SWA: median, 3.9 Hz; first and third quartiles, 2.4 and 37.3 Hz; *n* = 6, *z* = 0.10, *p* = 0.92, Wilcoxon matched-pairs test) or was significantly lower during the activation compared with the SWA state (theta-OFF neurons: 10%, 20 of 197; activation: median, 6.5 Hz; first and third quartiles, 3.5 and 16.6 Hz; SWA: median, 14.9 Hz; first and third quartiles, 6.3 and 22.2 Hz; *n* = 20, *z* = 2.80, *p* < 0.01, Wilcoxon matched-pairs test).

#### Firing of NI neurons in relation to the phase of hippocampal theta oscillation

**Figure 2.**
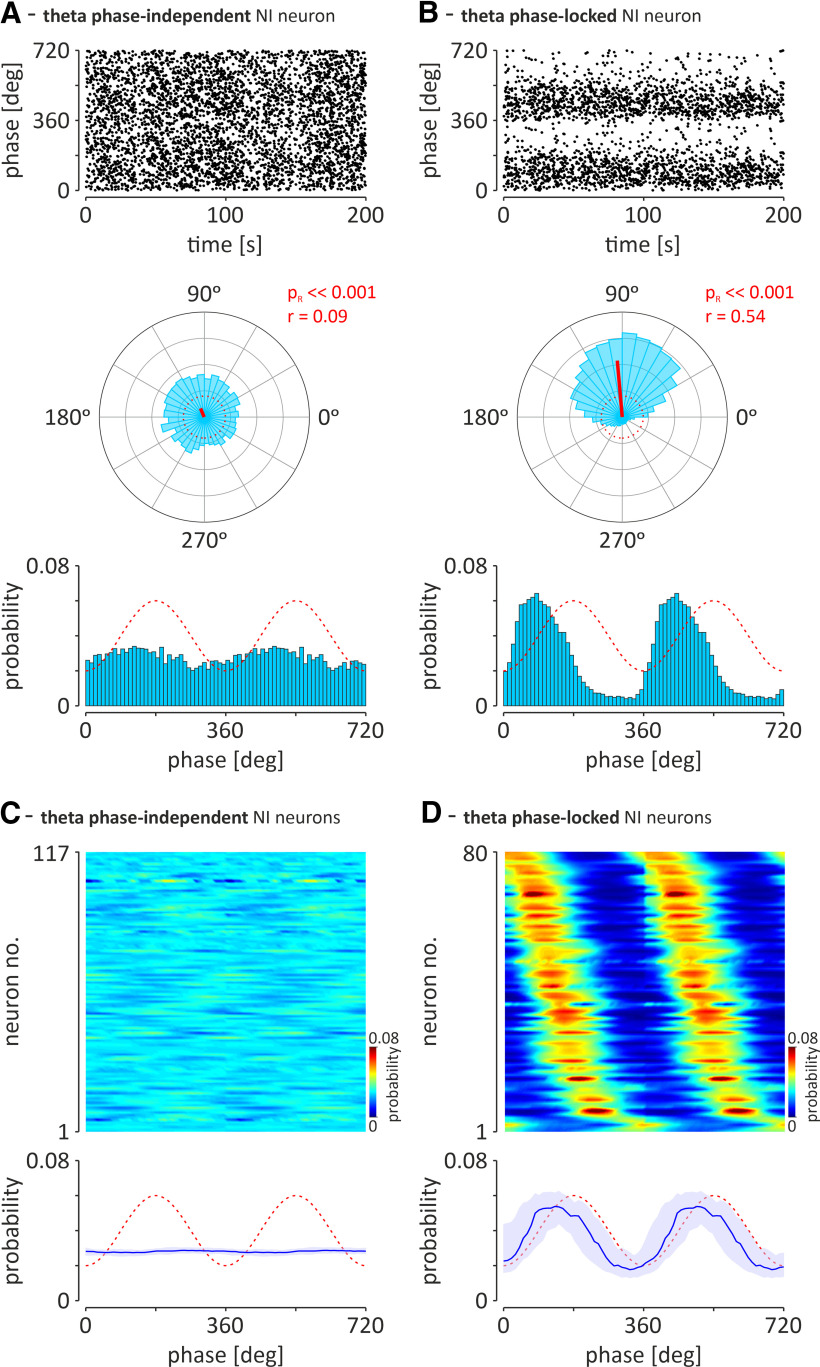
Two types of NI neurons distinguished on the basis of their preference to fire action potentials in a hippocampal theta phase-independent or theta phase-locked manner. ***A***, An exemplary NI neuron that fires action potentials in a theta phase-independent manner (*p*_R_ > 0.05 or *p*_R_ ≤ 0.05; *r* < 0.2). ***B***, An exemplary neuron with strong preference (*p*_R_ < 0.05; *r* ≥ 0.2) to fire during the specific phase of the ongoing hippocampal theta oscillation. Top, Scatter plots illustrating the position of individual action potentials (dots) around two cycles of the hippocampal theta oscillation. Middle, Circular plots illustrating the histogram of the angular distribution of action potentials around the cycle of the hippocampal theta oscillation. Red solid lines indicate direction vectors. The red, dotted circles indicate the length of the direction vector equal to 0.2. Bottom, Histograms illustrating the distribution of action potential firing probability around two cycles of hippocampal theta oscillation. The red, dashed line depicts idealized hippocampal theta oscillation. The trough of the hippocampal theta rhythm was defined as the zero phase (see Materials and Methods). Bins (all histograms) = 10°. In the case of both neurons, analysis of 200 s of firing recorded during cortical activation is shown. *r*, direction vector length; *p*_R_, confidence level of Rayleigh's test for uniformity. ***C***, ***D***, The preference to theta phase of all recorded theta phase-independent NI neurons and theta phase-locked NI neurons, respectively, during two theta cycles. Top, Heat maps of the probability to fire action potentials at a specific hippocampal theta phase for theta phase-independent (***C***; *n* = 117) and theta phase-locked (***D***; *n* = 80) neurons. The probability of action potential generation is color coded. The neurons are sorted according to the phase of the probability peak. Bottom, The median (blue line) phase preference of the two groups of NI neurons shown above. Light blue area indicates the interquartile range; red-dashed line illustrates idealized hippocampal theta oscillations.

Theta oscillations in the local field potential in the stratum lacunosum-moleculare of the CA1 region of the hippocampus, observed during brain activation, were the basis for determining whether NI neurons generate action potentials in a theta phase-locked manner. Fifty-nine percent of recorded NI neurons (117 of 197) fired action potentials in theta phase-independent manner [Fig. 2*A*,*C*; theta phase-independent neurons: *p*_R_ > 0.05, Rayleigh's test for uniformity; or direction vector length, *r* < 0.2 (at *p*_R_ ≤ 0.05)], whereas the firing of 41% of recorded NI neurons (80 of 197) was significantly locked with the phase of hippocampal theta oscillations (Fig. 2*B*,*D*; theta phase-locked neurons, *p*_R_ < 0.05, Rayleigh's test for uniformity; direction vector length, *r* ≥ 0.2). The population of theta phase-locked NI neurons, observed in our study, had a significant preference to fire action potentials during the rising phase of the hippocampal theta oscillation recorded from the hippocampal CA1 field (mean direction, 134.0 ± 7°; direction vector length, 0.55, at *p*_R_ < 0.001, *n* = 80). The phase preference of theta phase-locked NI neurons was not correlated either with their anteroposterior, lateromedial or dorsoventral position in the NI (*r* = 0.09, *p* = 0.35; *r* = 0.33, *p* = 0.07; and *r* = −0.09, *p* = 0.44, respectively; Zar, 1999).

#### Firing patterns of the NI neurons during hippocampal theta oscillation

In each group of NI neurons, both theta phase independent and theta phase locked, two additional subpopulations could be clearly differentiated, based on their pattern of electrical activity.

##### Theta phase-independent NI neurons.

**Figure 3.**
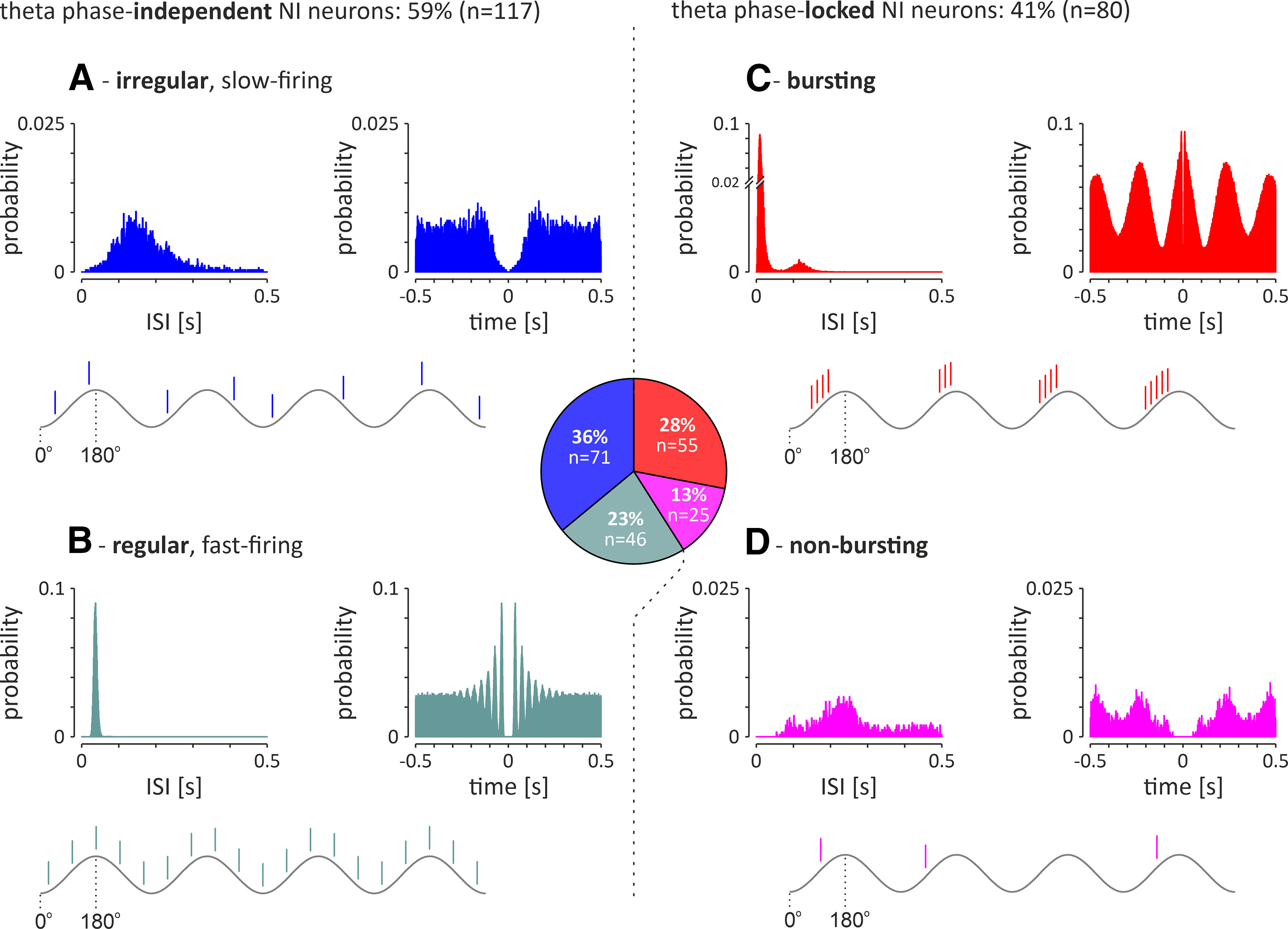
Electrophysiological types of recorded NI neurons (*n* = 197) recorded during cortical activation. Middle, Pie chart illustrating the proportion of the different types in the total NI neuron population. ***A***, ***B***, Left, Theta phase-independent neurons consist of the following two subgroups: irregular, slow-firing neurons (***A***) and regular, fast-firing neurons (***B***). ***C***, ***D***, Right, Theta phase-locked neurons consist of the following two subgroups: bursting neurons (***C***) and nonbursting neurons (***D***). In each panel, histograms of interspike intervals (left) and autocorrelation function plots (right) during cortical activation are shown for a representative neuron of each type. The theta-wave and action potentials generated by an idealized neuron of a given type are schematically presented below the histograms. Bins (all histograms), 10 ms.

In the population of theta phase-independent NI neurons (*n* = 117), the majority (61%, 71 of 117) were characterized by irregular, slow-firing action potentials (Fig. 3*A*), whereas a minority (39%, 46 of 117) were regular, fast-firing neurons (Fig. 3*B*). Regular, fast-firing NI neurons fired action potentials significantly faster and more regularly compared with the irregular, slow-firing neurons (Table 1).

**Table 1.** Firing of different types of NI neurons during state of cortical activation

			Median FR (Hz)	1st quartile; 3rd quartile (Hz)	Mann–Whitney test	CV	1st quartile; 3rd quartile	Mann–Whitney test
Cortical activation	Tph-indep.	Irregular, slow-firing (*n* = 71)	7.7	5.2; 17.6	*p* < 0.0001* U = 543.0	0.68	0.51; 0.9	*p* < 0.0001* U = 0.0
		Regular, fast-firing (*n* = 46)	26.5	16.0; 37.4		0.19	0.17; 0.21	
	Tph-locked	Bursting (*n* = 55)	42.6	27.5; 59.7	*p* < 0.0001* U = 39.0	1.1	0.9; 1.34	*p* < 0.0001* U = 319.5
		Nonbursting (*n* = 25)	5.5	4.5; 11.6		0.8	0.7; 1.0	

Tph, Theta phase; indep., independent; FR, firing rate;

*, significant difference.

##### Theta phase-locked NI neurons.

**Figure 4.**
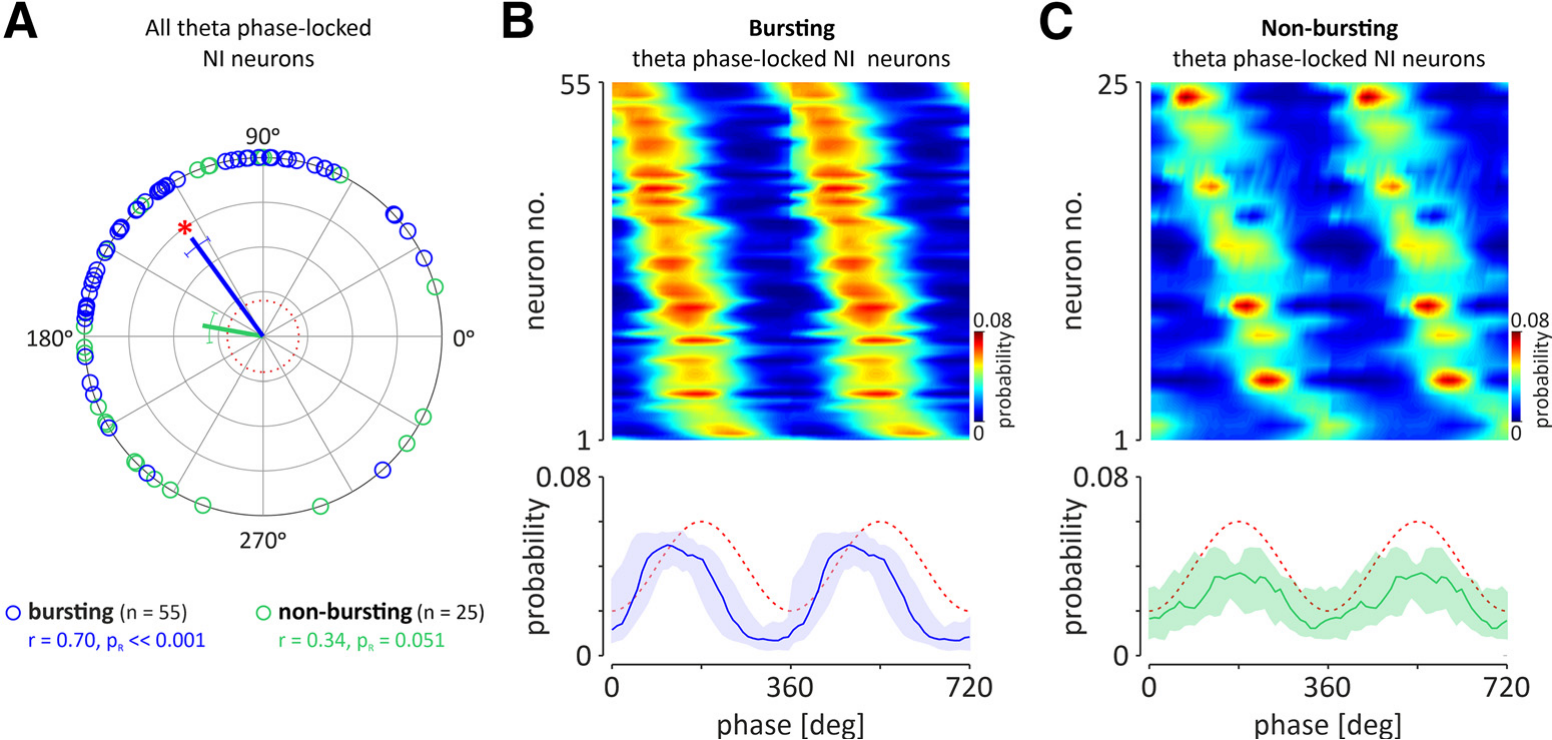
The preference of nonbursting and bursting, theta phase-locked NI neurons to fire action potentials at a specific phase of the hippocampal theta oscillation. ***A***, Circular plot illustrating the phase preference of individual bursting (blue; *n* = 55) and nonbursting (green; *n* = 25) neurons. Solid lines indicate the mean direction vector of a specific neuronal population (coded by color). Whiskers represent circular SEM directions. The red, dotted circle indicates the length of the direction vector equal to 0.2. Red asterisk, Significant phase preference of the population of theta bursting NI neurons; *r*, direction vector length; *p*_R_, confidence level of Rayleigh's test for uniformity. ***A***, ***B***, Heat maps of the probability to fire action potentials at a specific hippocampal theta phase for theta phase-locked bursting (***B***) and theta phase-locked nonbursting (***C***) neurons. The neurons are sorted according to the phase of the probability peak. Bottom, The median (blue and green lines) phase preference of the two groups of NI neurons shown above. Light-colored area, interquartile range; red-dashed line, idealized hippocampal theta oscillations. The trough of the hippocampal theta rhythm was defined as the zero phase.

**Figure 5.**
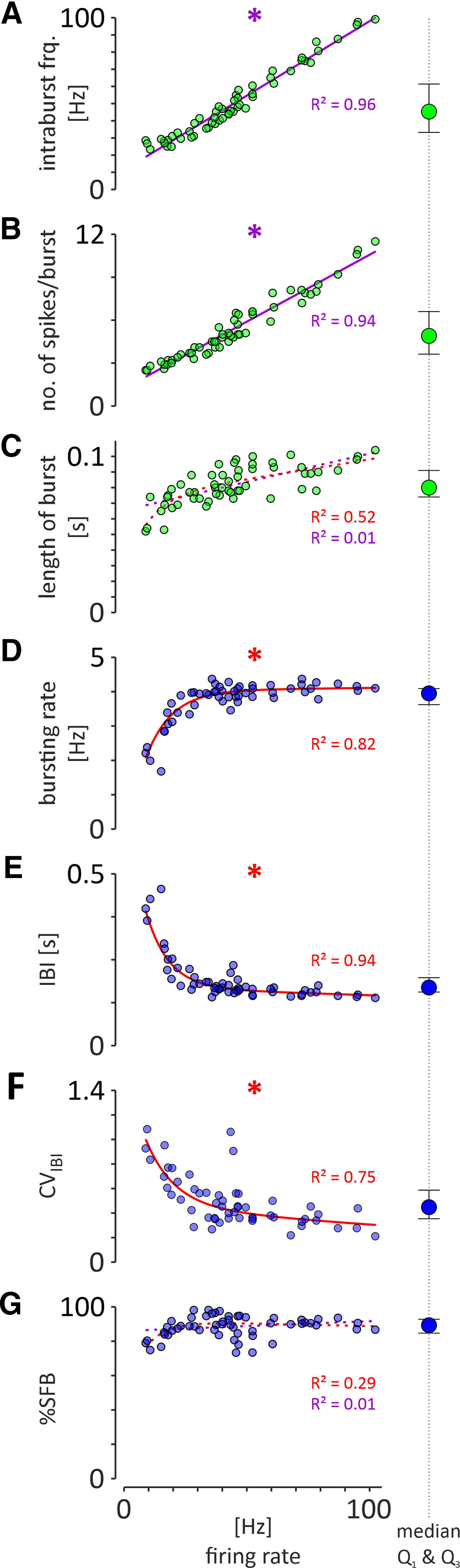
Correlation of the parameters describing the strength of the generated bursts (green circles). ***A***, Intraburst frequency. ***B***, Number of spikes per burst. ***C***, Length of burst, and the intensity of bursting (blue circles). ***D***, Bursting rate. ***E***, Interburst interval (IBI). ***F***, CV of IBI (CV_IBI_). ***G***, Percentage of spikes fired in bursts (%SFB), with the median firing rate of NI theta-bursting neurons (*n* = 55). Asterisks and fitted curves, drawn with a solid line, indicate a significant correlation (*R*^2^ ≥ 0.7), whereas fitted curves, drawn with a dotted line, indicate a non-significant correlation (*R*^2^ < 0.7). Individual *R*^2^ values are given on each plot. The type of fit used is color coded, as follows: linear, violet; exponential, red. Note that in the case of non-significant correlations, linear and exponential fits were performed. The blue circles with whiskers on the side of each graph illustrate the median, and first and third quartiles (Q1 and Q3) of the analyzed parameters.

In the theta phase-locked group of NI neurons (*n* = 80), the majority (69%, 55 of 80) generated bursts of action potentials, as indicated by a bimodal distribution of interspike intervals and autocorrelation function plots with first-order and second-order peaks (Fig. 3*C*). A smaller proportion of theta phase-locked NI neurons (31%, 25 of 80) generated a nonbursting pattern of electrical activity (Fig. 3*D*). Bursting, theta phase-locked NI neurons fired action potentials significantly faster compared with the nonbursting, theta phase-locked neurons (Table 1). The vast majority of theta phase-locked neurons was located in the NI pars dissipata [Fig. 1*C*,*D*; laterality > 200 µm; i.e., all bursting neurons (100%, 55 of 55) and all but two nonbursting neurons (92%, 23 of 25)]. The population of theta-bursting NI neurons had a strong preference to fire action potentials during the rising phase of the hippocampal theta oscillation (Fig. 4*A*,*B*; mean direction, 126.0 ± 6.8°; direction vector length, 0.70; *p*_R_ < 0.001; *n* = 55). In contrast, nonbursting, theta phase-locked NI neurons, had uniformly distributed phase preferences, and, thus, this population of NI neurons displayed no significant preference to fire action potentials at a particular phase of the hippocampal theta oscillation (Fig. 4*A*,*C*; mean direction, 169.8 ± 16.8°; direction vector length, 0.34; *p*_R_ = 0.051; *n* = 25). Analysis of the correlation of parameters describing the strength of generated bursts and intensity of bursting with firing of NI theta-bursting neurons revealed that intraburst frequency and the number of spikes generated per burst were positively, linearly correlated with the firing rate of a neuron (*R*^2^ = 0.96 and *R*^2^ = 0.94, respectively; Fig. 5*A*,*B*), while the burst length did not display a significant correlation (linear, *R*^2^ = 0.01; exponential, *R*^2^ = 0.52; Fig. 5*C*). At the same time, bursting rate, interburst interval, and coefficient of variation (CV) of interburst interval were exponentially correlated with the firing rate (positive correlation, *R*^2^ = 0.82; negative correlations: *R*^2^ = 0.94 and *R*^2^ = 0.75, respectively; Fig. 5*D–F*), while the percentage of spikes generated in bursts did not show a significant correlation (neither linear, *R*^2^ = 0.01; nor exponential, *R*^2^ = 0.29; Fig. 5*G*).

#### Cross-correlation of NI neuronal firing during hippocampal theta oscillation

**Figure 6.**
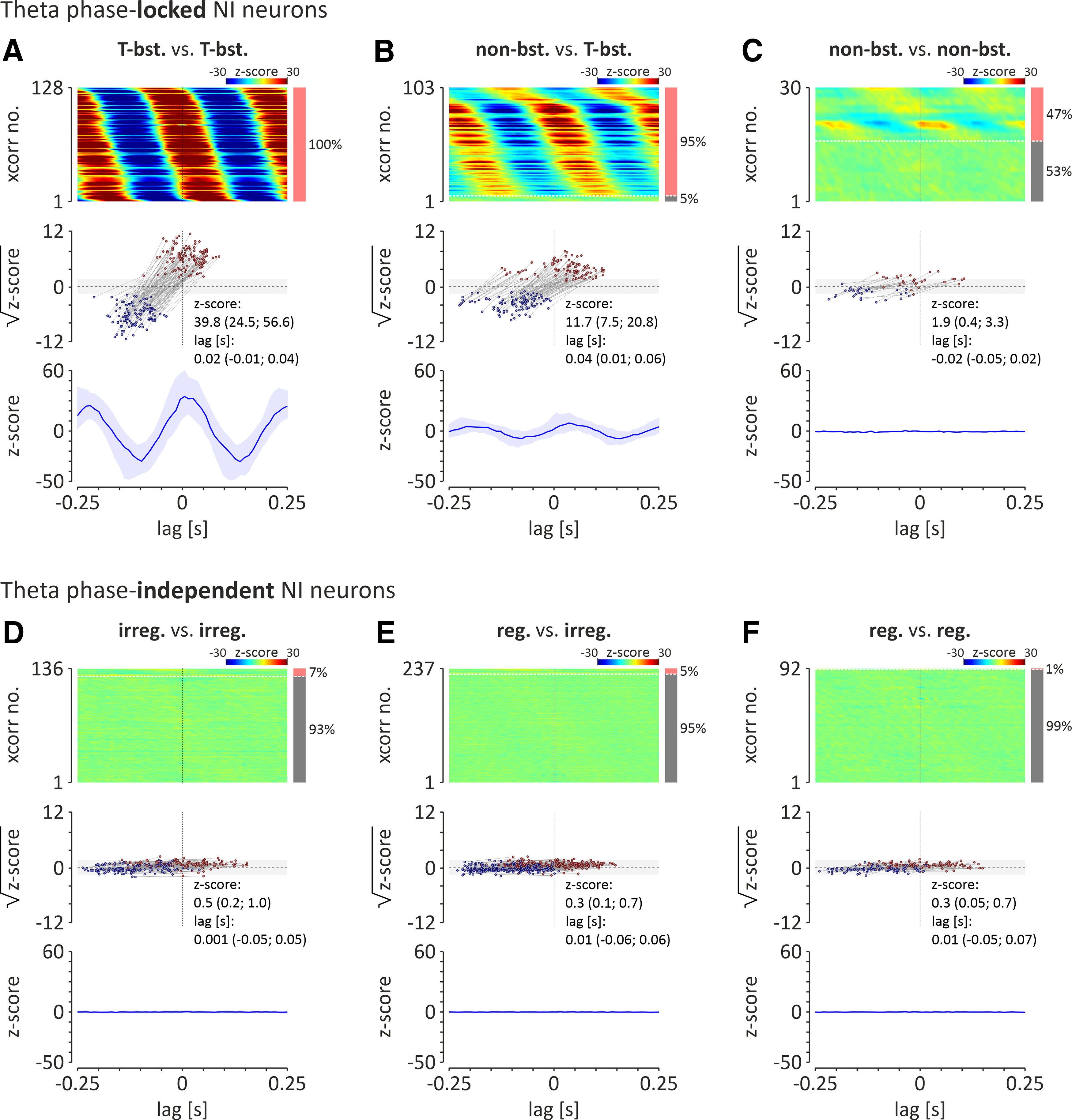
***A–F***, Cross-correlation analysis of spontaneous activity, observed during cortical activation, of theta phase-locked NI neurons: theta bursting versus theta bursting (T-bst. vs T-bst.; ***A***); nonbursting versus theta bursting (nonbst. vs T-bst.; ***B***); nonbursting versus nonbursting (non-bst. vs non-bst.; ***C***); and theta phase-independent NI neurons: irregular firing versus irregular firing (irreg. vs irreg.; ***D***); regular firing versus irregular firing (reg. vs irreg.; ***E***); regular firing versus regular firing (reg. vs reg.; ***F***). Top, Heatmaps showing cross-correlations of activity of NI theta bursting (T-bst.) neuron pairs. Cross-correlations are sorted by the time of the peak closest to zero-lag. Cross-correlation with significant peak values (*z* score > 2.575; >99% confidence level) are shown first (from top). The bar on the right indicates the proportion of pairs of neurons with activity correlated (light red) and uncorrelated (gray). Note that all theta-bursting neuronal pairs were significantly cross-correlated. Middle, Times and square roots of *z* score values of the cross-correlogram positive peaks (closest to zero-lag; red dots), and preceding it, negative peaks (blue dots). Light gray area indicates the range of non-significant *z* score values (range, ±2.575; equivalent to ±99% confidence limits). Bottom, The median (blue line) of all cross-correlograms shown above. Light blue area indicates interquartile range.

**Figure 7.**
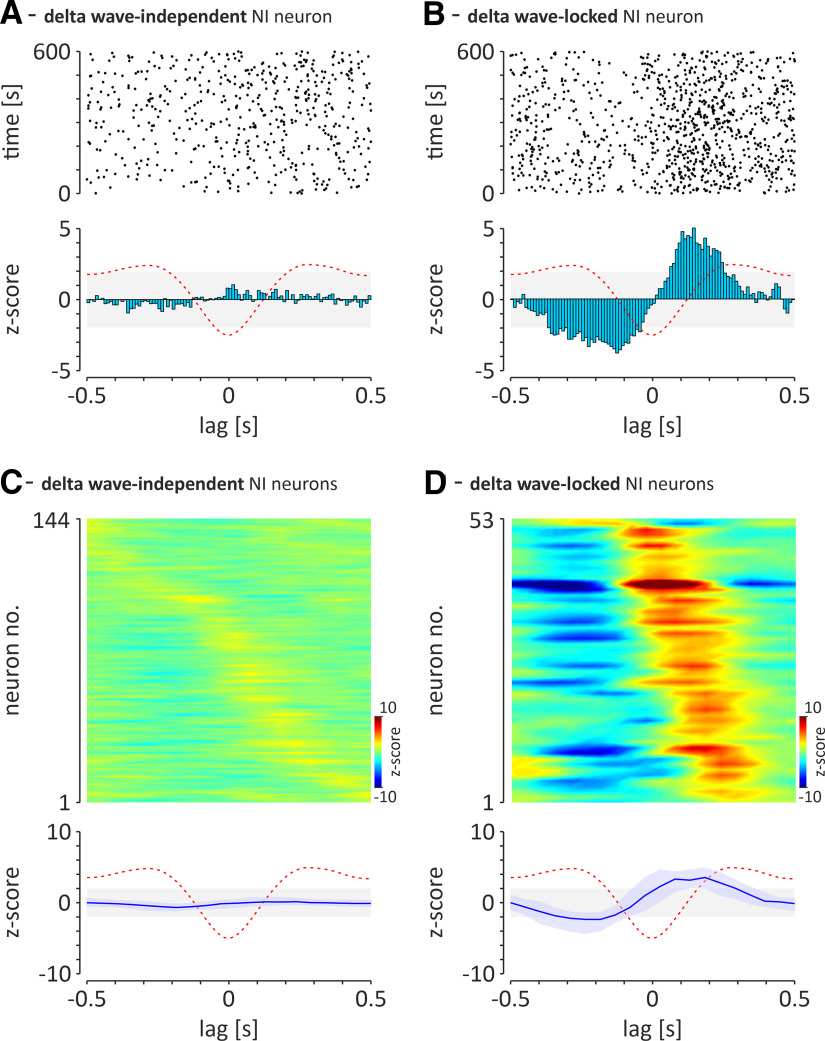
Two types of NI neurons that can be distinguished on the basis of their preference to fire action potentials in a hippocampal delta wave-independent or delta wave-locked manner. ***A***, An exemplary NI neuron that fires action potentials in a delta wave-independent manner (peak *z* score of delta wave zero-phase-triggered PSTH < 1.96). ***B***, An exemplary neuron with strong preference to fire in a hippocampal delta wave-locked manner (peak *z* score of delta wave zero phase-triggered PSTH peak, ≥1.96). Top, Scatter plots illustrating the position of individual action potentials (dots) around the delta wave-negative peak. Bottom, Histograms illustrating the distribution of action potential firing around the delta wave-negative peak. The red-dashed line, Idealized hippocampal delta waves; light gray area indicates the range of non-significant *z* score values (range, ±1.96; equivalent to ±95% confidence limits). Bins, 10 ms. ***C***, ***D***, The relationship between firing of all recorded delta wave-independent (***C***) and delta wave-locked (***D***) NI neurons and the negative peak of hippocampal CA1 delta waves. Top, Heat maps illustrating *z*-score values of delta wave zero phase-triggered PSTH (10 ms bins) for delta wave-independent (***C***; *n* = 144) and delta wave-locked (***D***; *n* = 53) neurons. The *z* score values are color coded. Neurons are sorted according to the time of the *z*-value peak. Bottom, The median (blue line) delta wave zero phase-triggered PSTH of the two groups of NI neurons shown above. Light blue area, interquartile range; red-dashed line, illustration of idealized hippocampal delta wave.

A cross-correlation analysis of activity in pairs of simultaneously recorded neurons (during cortical activation), belonging to the same or different populations of NI neurons (as described above), was performed (Fig. 6), revealing that firing of all theta bursting NI neurons was highly synchronized (100%, 128 of 128 pairs of neurons; peak *z* score > 2.575; median peak *z* score = 39.8; first and third quartiles: 24.5 and 56.6; median peak time = 0.02 s; first and third quartile: −0.01 and 0.04; Fig. 6*A*). Similarly, the majority of nonbursting, theta phase-locked neurons fired in synchrony with theta bursting neurons (95%, 98 of 103 pairs of neurons; peak *z* score > 2.575; median peak *z* score = 11.7; first and third quartiles: 7.5 and 20.8; median peak time = 0.04 s; first and third quartiles: 0.01 and 0.06; Fig. 7*B*). Less than half of the analyzed pairs of nonbursting, theta phase-locked neurons was synchronized (47%, 14 of 30 pairs of neurons; peak *z* score > 2.575) which resulted in non-significant synchrony within this population of NI neurons (median peak *z* score = 1.9; first and third quartiles: 0.4 and 3.3; median peak time = −0.02 s; first and third quartiles: −0.05 and 0.02; Fig. 6*C*). At the same time, a vast majority of analyzed pairs of theta phase-independent NI neurons displayed asynchronous firing (cross-correlation peak *z* score < 2.575): 93% (127 of 136) of paired irregular firing neurons (Fig. 6*D*); 95% (225 of 237) of paired regular and irregular firing neurons (Fig. 6*E*); and 99% (91 of 92) of paired regular firing neurons (Fig. 6*F*).

#### Firing of NI neurons in relation to the delta waves during SWA

Slow-wave activity observed in the local field potential in the CA1 region of the hippocampus was the basis for determining whether NI neurons generate action potentials in a delta wave-dependent manner. A majority of recorded NI neurons (73%; 144 of 197) fired action potentials independently of delta waves [Fig. 7*A*,*C*; delta wave-independent neurons; peak *z* score of delta-wave, zero phase-triggered peristimulus time histogram (PSTH) < 1.96], whereas the firing of 27% of recorded NI neurons (53 of 197) was significantly locked with the hippocampal delta waves (Fig. 7*B*,*D*; delta wave-locked neurons; peak *z* score of delta-wave zero-phase triggered PSTH peak ≥ 1.96). The latter population of neurons displayed the highest probability of generating action potentials on the ascending phase of the delta wave (i.e., after its negative peak; median lag = 0.15 s; first and third quartiles: 0.09 and 0.21 s). Of the 144 delta wave-independent NI neurons, the majority (69%, 100 of 144) were neurons that, during cortical activation, were also theta phase independent (85% of the theta phase-independent population; 100 of 117). The remaining 44 delta wave-independent NI neurons (31%) were theta phase locked (55% of the theta phase-locked population, 44 of 80) during cortical activation. Of the 53 delta wave-locked NI neurons, the majority (68%, 36 of 53 neurons) were neurons that, during cortical activation, were theta phase locked (45% of the theta phase-locked population, 36 of 80 neurons). The remaining 17 (32%) delta wave-locked NI neurons, during cortical activation were theta phase independent (15% of the theta phase-independent population, 17 of 117 neurons).

#### Firing patterns of the NI neurons during hippocampal SWA

In each group of NI neurons, both delta wave independent and delta wave locked, two additional subpopulations could be clearly differentiated, based on their pattern of electrical activity.

##### Delta wave-independent NI neurons.

**Figure 8.**
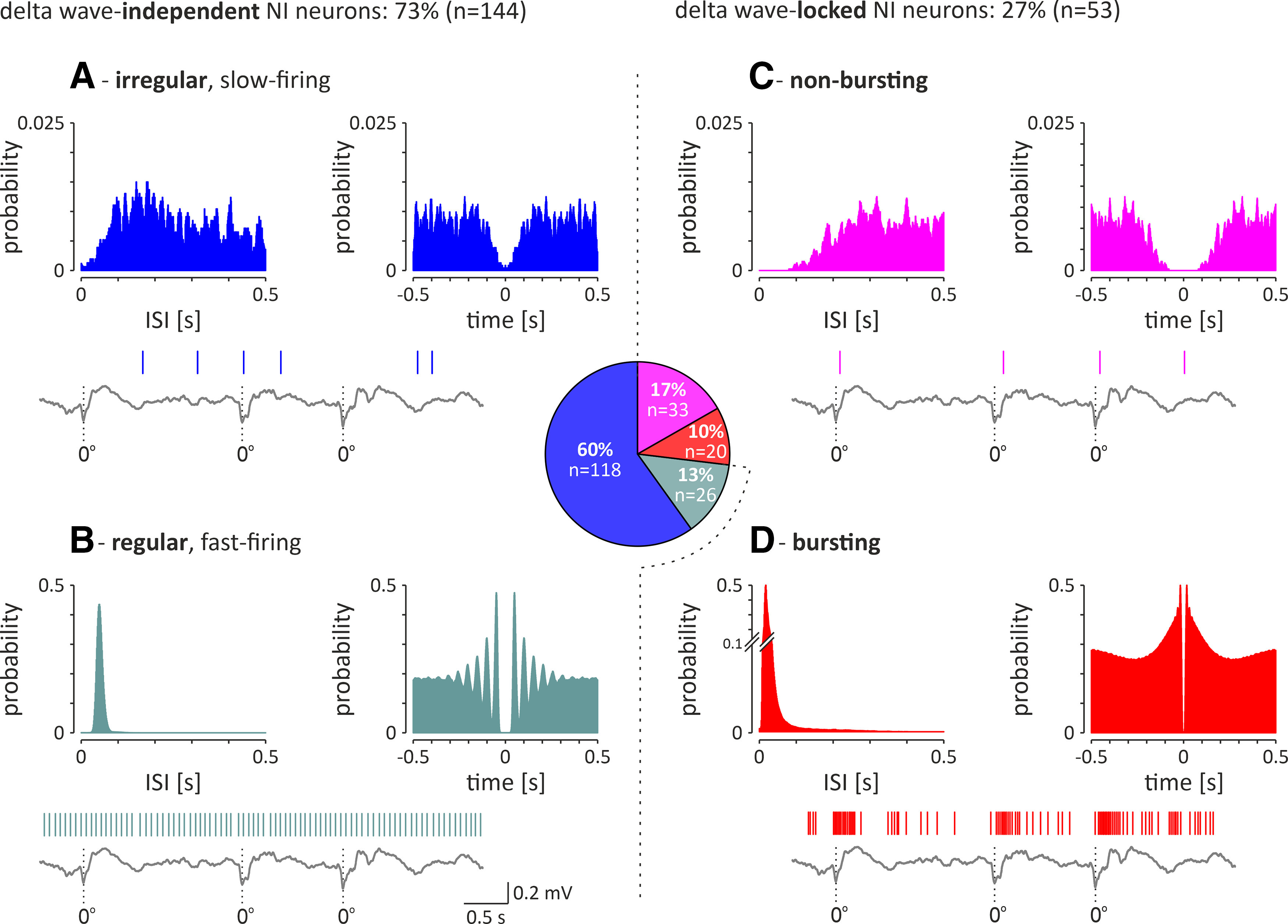
Electrophysiological types of recorded NI neurons (*n* = 197) recorded during SWA. Middle, Pie chart illustrating the proportion of the different types in the total NI neuron population. ***A***, ***B***, Left, Delta wave-independent neurons consist of the following two subgroups: irregular, slow-firing neurons (***A***) and regular, fast-firing neurons (***B***). ***C***, ***D***, Right, Delta wave-locked neurons consist of the following two subgroups: nonbursting neurons (***C***) and bursting neurons (***D***). In each panel, histograms of interspike intervals (left) and autocorrelation function plots (right) during hippocampal SWA are shown for a representative neuron of each type. A fragment of hippocampal LFP is shown at the bottom of each panel. Vertical lines indicate action potentials generated by simultaneously recorded different types of NI neurons. Bins (all histograms), 10 ms.

In the population of delta wave-independent NI neurons (*n* = 144), the vast majority (82%, 118 of 144 neurons) were characterized by irregular, slow firing of action potentials (Fig. 8*A*), whereas a minority (18%, 26 of 118 neurons) were regular, fast-firing neurons (Fig. 8*B*). Regular, fast-firing NI neurons fired action potentials significantly faster and more regularly than the irregular, slow-firing neurons (Table 2).

**Table 2.** Firing of different types of NI neurons during state of cortical SWA

			Median FR (Hz)	1st quartile; 3rd quartile (Hz)	Mann–Whitney test	CV	1st quartile; 3rd quartile	Mann–Whitney test
Cortical SWA	Dw-indep.	Irregular, slow-firing (*n* = 118)	6.5	3.0; 12.6	*p* < 0.0001* U = 317.0	1.0	0.84; 1.4	*p* < 0.0001* U = 0.0
		Regular, fast-firing (*n* = 26)	29.4	24.1; 35.2		0.17	0.15; 0.19	
	Dw-locked	Bursting (*n* = 20)	20.8	17.4; 33.1	*p* < 0.0001* U = 106.0	1.1	1.0; 1.2	*p* = 0.012 *U* = 194.0
		Nonbursting (*n* = 33)	9.0	3.8; 15.5		0.9	0.76; 1.1	

Dw, Delta wave; indep., independent; FR, firing rate

*, significant difference.

##### Delta wave-locked NI neurons.

In the delta wave-locked group of NI neurons (*n* = 53), the majority (62%, 33 of 53 neurons) generated a nonbursting pattern of electrical activity (Fig. 8*C*). A smaller proportion of delta wave-locked NI neurons (38%, 20 of 53) generated bursts of action potentials, as indicated by a distribution of interspike intervals (a dominant peak at short intervals and a substantially smaller and slowly decaying count of longer intervals) and an autocorrelation plot with a first-order peak sharply centered around zero (Fig. 8*D*). Bursting, delta wave-locked NI neurons fired action potentials significantly faster than the nonbursting, delta wave-locked neurons (Table 2).

#### Cross-correlation of NI neuronal firing during SWA

**Figure 9.**
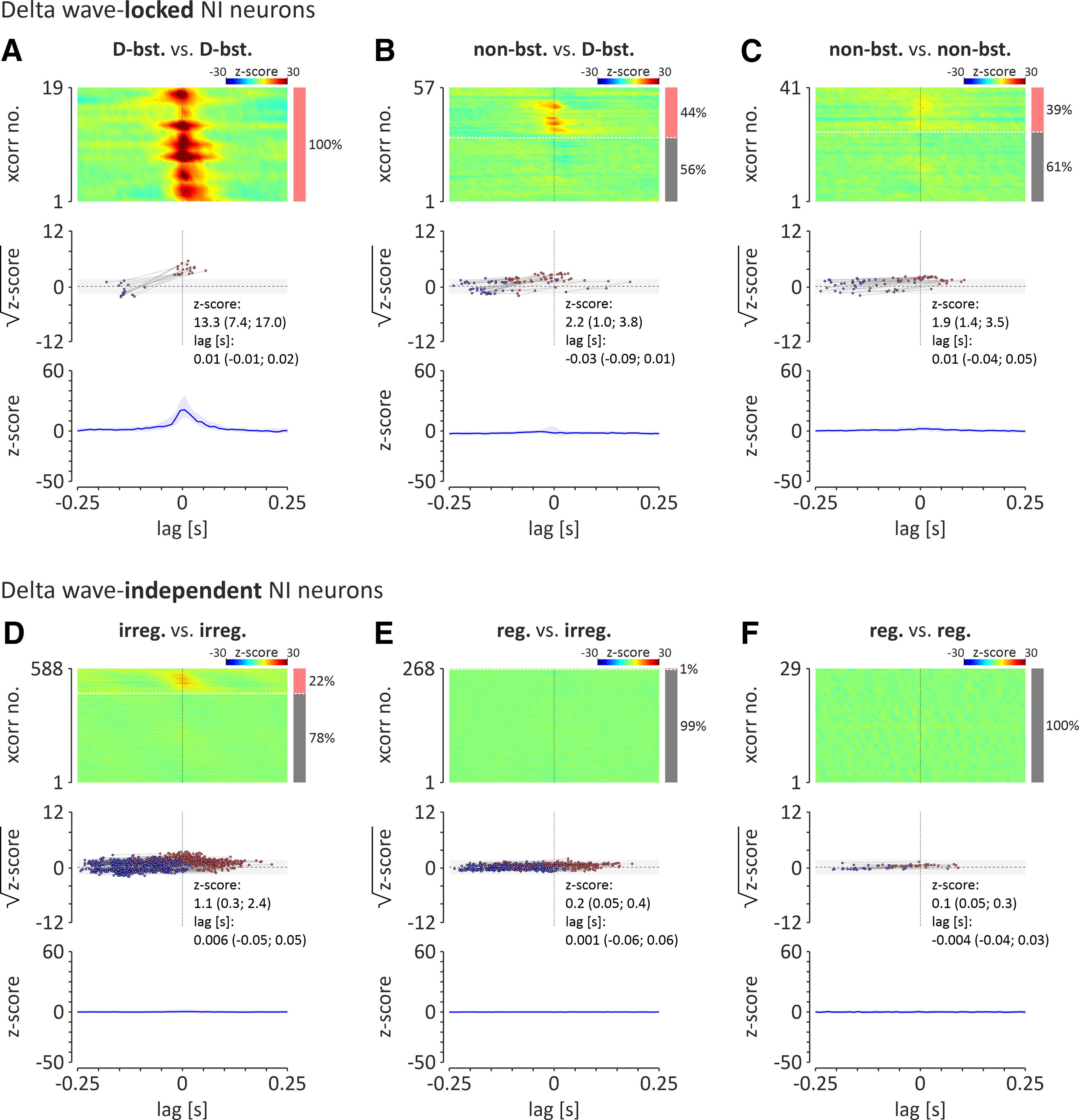
Cross-correlation analysis of spontaneous activity, observed during cortical SWA, of delta wave-locked NI neurons [delta bursting vs delta bursting (D-bst. vs D-bst.; ***A***); nonbursting versus delta bursting (non-bst. vs D-bst.; ***B***); nonbursting versus nonbursting (non-bst. vs non-bst.; ***C***); and delta wave-independent NI neurons irregular firing versus irregular firing (irreg. vs irreg.; ***D***); regular firing versus irregular firing (reg. vs irreg.; ***E***); and regular firing versus regular firing (reg. vs reg.; ***F***). Top, Heatmaps illustrating cross-correlations of activity of NI theta bursting (T-bst.) neuron pairs. Cross-correlations are sorted by the time of the peak closest to zero-lag. Cross-correlation with significant peak values (*z* score > 2.575; >99% confidence level) are shown first (from top). The bar on the right indicates the proportion of pairs of neurons whose activity was correlated (light red) and uncorrelated (gray). Note that all theta-bursting neuronal pairs were significantly cross-correlated. Middle, Times and square roots of *z*-score values of the positive peaks of cross-correlograms (closest to zero-lag; red dots) and preceding it negative peaks (blue dots). Light gray area indicates the range of non-significant *z*-score values (range, ±2.575; equivalent to ±99% confidence limits). Bottom, median (blue line) of all cross-correlograms shown above. Light blue area indicates interquartile range.

A cross-correlation analysis of activity in pairs of simultaneously recorded neurons (during cortical SWA), belonging to the same or different populations of NI neurons (as described above), was performed (Fig. 9) and revealed that the firing of all delta-bursting NI neurons is highly synchronized (100%, 19 of 19 pairs of neurons; peak *z* score > 2.575; median peak *z* score = 13.3; first and third quartile: 7.4 and 17.0; median peak time = 0.01 s, first and third quartile: −0.01 and 0.02; Fig. 9*A*). Less than half of nonbursting, delta wave-locked neurons fired in synchrony with delta-bursting neurons (44%, 25 of 57 pairs of neurons; peak *z* score > 2.575), which resulted in non-significant synchrony between these two populations of NI neurons (median peak *z* score = 2.2; first and third quartiles: 1.0 and 3.8; median peak time = −0.03 s; first and third quartiles: −0.09 and 0.01; Fig. 9*B*). Similarly, only 39% (16 of 41) of analyzed pairs of nonbursting, delta wave-locked neurons were synchronized (peak *z* score > 2.575), which resulted in non-significant synchrony within this population of NI neurons (median peak *z* score = −0.03; first and third quartile: −0.09 and 0.01; median peak time = 0.01 s; first and third quartiles: −0.04 and 0.05; Fig. 9*C*). At the same time, a vast majority of analyzed pairs of delta wave-independent NI neurons displayed asynchronous firing (cross-correlation peak *z* score < 2.575): 78% (459 of 588) of paired irregular firing neurons (Fig. 9*D*), 99% (265 of 268) of paired regular and irregular firing neurons (Fig. 9*E*), and all (29 of 29) of paired regular firing neurons (Fig. 9*F*).

### Responses of NI neurons to input form the MS

#### Responses of the NI neurons to optogenetic stimulation of the MS *in vivo*

**Figure 10.**
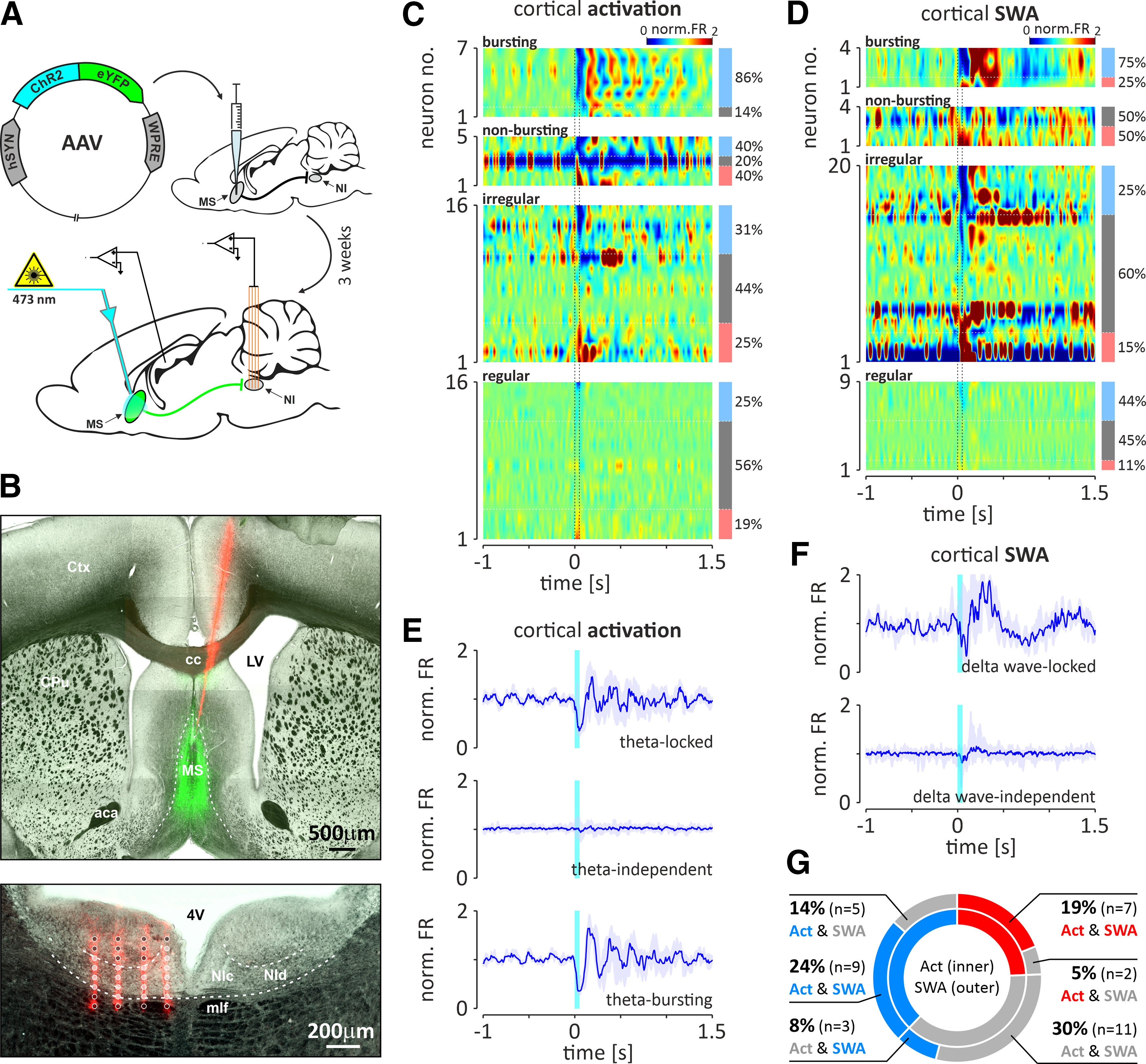
Responses of different types of NI neurons to the optogenetic stimulation of the MS during hippocampal theta and SWA. ***A***, Schematic illustrating the injection of viral vector into the MS and the experimental procedure conducted 3 weeks after the injection. ***B***, Histologic verification of the positioning of the optical fiber above the MS and the recording MEA in the NI. Top, An example of the DiI-filled trace (red) of the optical fiber terminating above the MS transfected with the viral vector (green). aca, Anterior commissure, anterior part; cc, corpus callosum; Ctx, cerebral cortex; CPu, caudate putamen; LV, lateral ventricle. Bottom, An example of the DiI-filled traces (red) of the four shanks of the MEA. Light gray spots represent electrodes positioned within the NI, whereas dark gray spots are localized outside the NI. NIc, NI pars compacta; NId, NI pars dissipata; 4V, fourth ventricle; Cb, cerebellum; DTg, dorsal tegmental nucleus; mlf, medial longitudinal fasciculus. ***C***, ***D***, Heatmaps illustrating the responses of different types of NI neurons to MS stimulation during cortical activation and SWA, respectively. The values of PSTHs (10 ms bins; normalized to baseline) are color coded. Vertical dotted lines indicate MS stimulation onset and offset (laser light: 473 nm, <10 mW, 50 ms). Light blue, bright red, and gray bars to the right of the heatmaps indicate the proportion of inhibited, excited, and nonresponding neurons, respectively. ***E***, ***F***, Response of selected populations of NI neurons to MS stimulation during cortical activation and SWA, respectively. Responses of theta-locked, theta-independent, theta-bursting, delta wave-locked, and delta wave-independent populations of NI neurons are shown. Blue line, Median PSTH value; light blue area, interquartile range; cyan bar, stimulation time. ***G***, Pie chart illustrating the proportions of response types of NI neurons with responses to MS stimulation in both cortical states (blue, inhibition; red, excitation; gray, no response).

**Figure 11.**
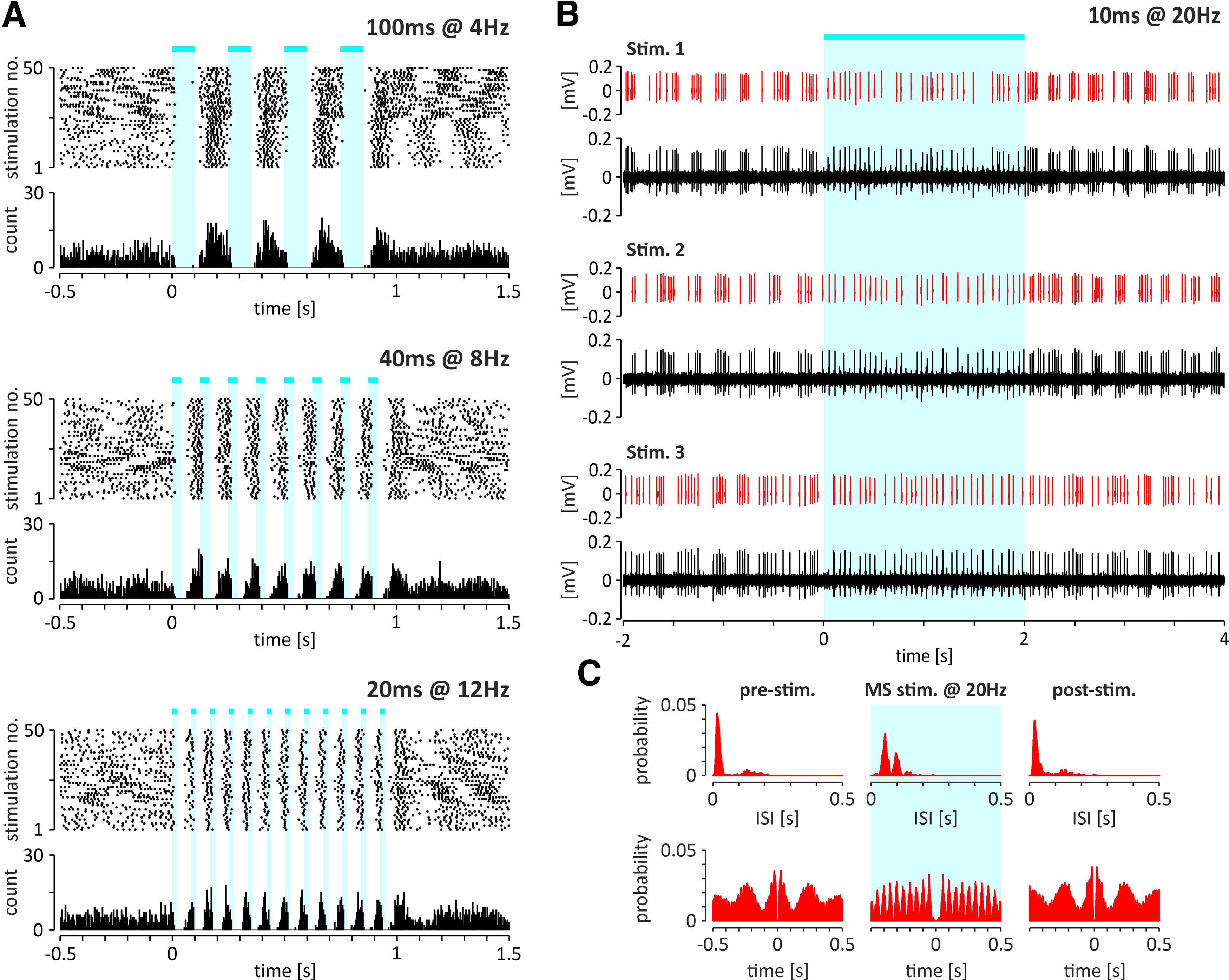
***A***, Scatter plots (top) and histograms (bottom) illustrating the exemplary response of a theta-bursting NI neuron to an optogenetic stimulation at three different theta frequencies (4, 8, and 12 Hz). Note that in all cases the neuron reacted with inhibition followed by a rebound excitation. ***B***, Responses of the same NI theta-bursting neuron to high-frequency stimulation of the MS (20 Hz). Representative fragments of the raw signal and separated spikes (red) of the recorded NI neuron. ***C***, Histograms of interspike intervals (top) and autocorrelation function plots (bottom) of an exemplary theta-bursting NI neuron (shown on ***A*** and ***B***) before (left), during (middle; marked with light blue color), and after (right) the high-frequency, optogenetic stimulation of the MS. Cyan-colored bars indicate the laser light (473 nm, <10 mW) pulses. Bins (all histograms), 10 ms.

The effective transfection of the MS [AAV2-hSyn-hChR2(H134R)-EYFP] as well as the position of the multielectrode array within the NI and the optical fiber above the MS were confirmed in four rats (Fig. 10*A*,*B*). The spontaneous activity of 63 NI neurons (neurons/rat: average, *n* = 16; range, *n* = 7–28) and their responses to the optogenetic stimulation of the MS were recorded. Responses of 37 neurons were recorded during both cortical activation and SWA, and the remaining neurons during only one of the cortical states. During cortical activation, the majority (86%, 6 of 7) of bursting theta phase-locked neurons were inhibited by MS stimulation (Fig. 10*C*,*E*, Table 3), and the cessation of each light pulse-evoked inhibition was followed by rebound burst of action potentials. Theta phase-locked nonbursting neurons responded to MS activation with excitation and inhibition in equal proportions (40%, 2 of 5 neurons; Fig. 10*C*,*E*, Table 3). It is noteworthy that only a minority of theta bursting neurons (14%, 1 of 7) and nonbursting neurons (20%, 1 of 5) did not respond to MS stimulation, and at population level, theta phase-locked NI neurons displayed an inhibitory response followed by rebound excitation (Fig. 10*E*). Interestingly, MS stimulation with a series of pulses at frequencies in the theta band (4, 8, and 12 Hz) resulted in the synchronization of the theta-bursting NI neurons to stimulation frequency (Fig. 11*A*). Conversely, high-frequency stimulation of the MS disrupted the theta bursting of NI neurons (Fig. 11*B*,*C*). On the other hand, 56% (9 of 16) and 44% (7 of 16) of theta phase-independent regular, fast-firing neurons and irregular, slow-firing neurons, respectively, did not respond to MS optogenetic stimulation (Fig. 10*C*, Table 3). At the same time, excitatory and inhibitory responses were observed in a similar proportion in both theta-independent neuron populations (Fig. 10*C*, Table 3), resulting in no response at the population level (Fig. 10*E*). During cortical SWA, stimulation of the MS inhibited a majority of delta-bursting NI neurons, while among nonbursting delta wave-locked cells, only excitatory responses were observed (Fig. 10*D*, Table 3). However, because of the small number of recorded neurons, it was not possible to conclusively determine whether, if at all, delta wave-locked neurons display a dominant response at the population level (Fig. 10*F*). In contrast, delta wave-independent neurons, although more frequently responsive to MS stimulation with inhibition than excitation (25% vs 15% and 44% vs 11%, respectively, for irregular and regular firing neurons; Fig. 10*D*, Table 3), did not display a dominant response at the population level (Fig. 10*F*). Among NI neurons for which responses to MS stimulation were observed in both cortical states (*n* = 37), 19% (7 of 32) were excited, 24% (9 of 37) were inhibited, and 30% (11 of 37) did not respond in both cortical activation and SWA states (Fig. 10*G*). A relatively small fraction of NI neurons responded to MS stimulation in only one cortical state, and none changed the direction of the response between cortical states (Fig. 10*G*).

**Table 3.** Parameters of responses of NI neurons to the MS optogenetic stimulation

			Response type	Fraction (count)	FR change (%)	Latency to maximum (ms)	Duration (ms)
Cortical activation	Tph-locked	Bursting (*n* = 7)	Inhibitory	86% (6)	−45 (−53; −41)	42.5 (35; 85.3)	120 (107; 151.5)
			Excited	0%			
			No response	14% (1)			
		Nonbursting (*n* = 5)	Inhibitory	40% (2)	−65	57	142.5
			Excited	40% (2)	76	46	104.5
			No response	20% (1)			
	Tph-indep.	Irregular (*n* = 16)	Inhibitory	31% (5)	−58 (−71; −25)	68 (34.5; 91)	131 (87.5; 188)
			Excited	25% (4)	63 (43; 99)	51.5 (47.8; 102.5)	154 (99.8; 273.5)
			No response	44% (7)			
		Regular (*n* = 16)	Inhibitory	25% (4)	−12 (−32; −5)	38.5 (25.3; 51.8)	126 (106.8; 139.3)
			Excited	19% (3)	25 (11; 33)	29 (23; 46)	118 (103; 123)
			No response	56% (9)			
Cortical SWA	Dw-locked	Bursting (*n* = 4)	Inhibitory	75% (3)	−74 (−74; −54)	92 (48; 109)	132 (110; 140)
			Excited	25% (1)	74	63	128
			No response	0%			
		Nonbursting (*n* = 4)	Inhibitory	0%			
			Excited	50% (2)	77	40.5	173
			No response	50% (2)			
	Dw-indep.	Irregular (*n* = 20)	Inhibitory	25% (5)	−63 (−75; −33)	46 (38; 56)	108 (105.5; 125.5)
			Excited	15% (3)	104 (102; 106)	54 (36; 123)	121 (103; 294)
			No response	60% (12)			
		Regular (*n* = 9)	Inhibitory	44% (4)	−17 (−20; −7)	48 (30.8; 63)	128 (79.3; 162.5)
			Excited	11% (1)	14	30	122
			No response	45% (4)			

Tph, Theta phase; indep., independent; Dw, delta wave; FR, firing rate.

#### Responses of NI neurons to optogenetic stimulation of the MS originating terminals—*ex vivo* recordings

**Figure 12.**
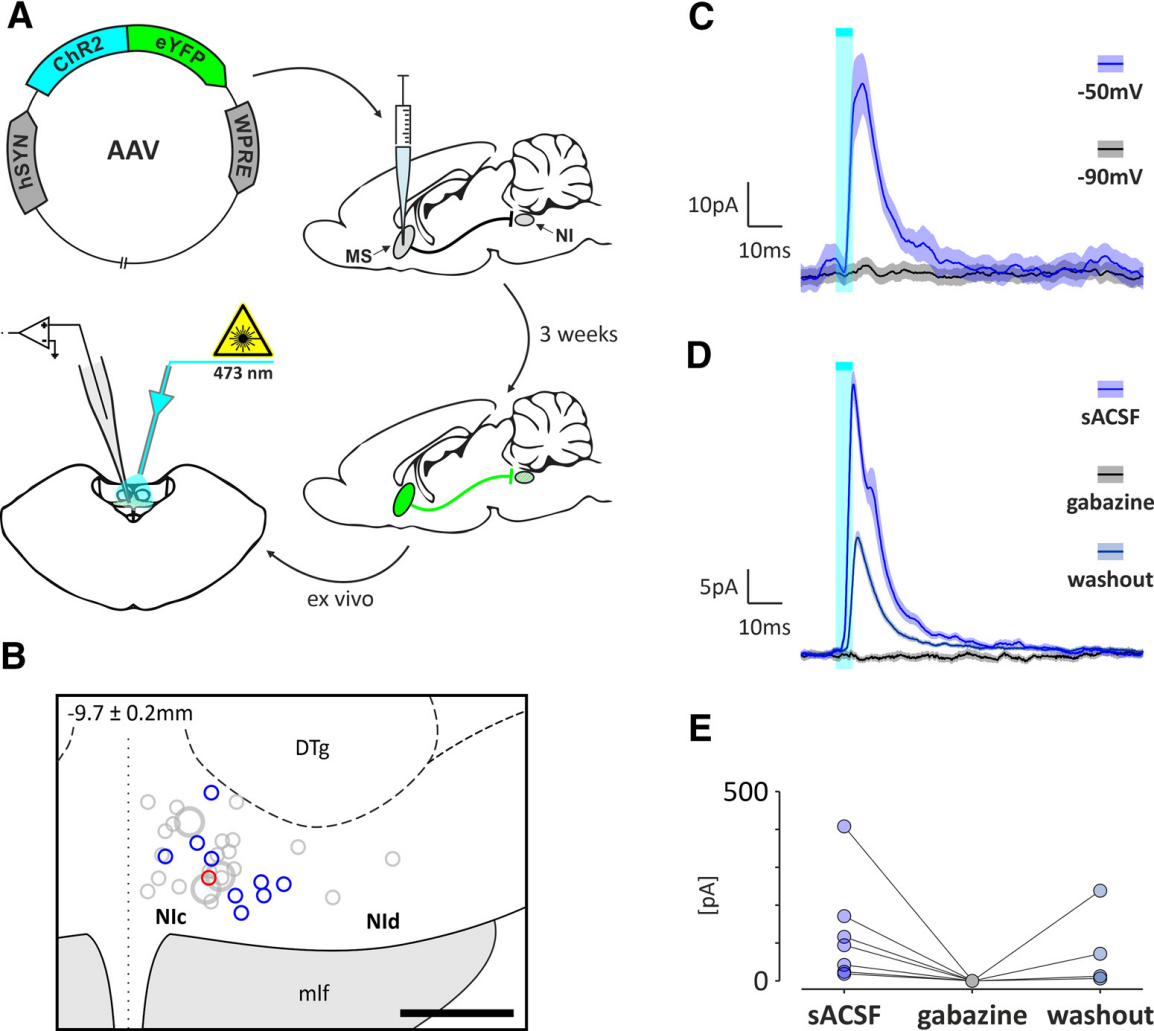
*Ex vivo* responses of NI neurons to optogenetic stimulation of MS originating axonal endings. ***A***, Schematic representation of the experimental design illustrating the viral vector injection procedure and subsequent MS axonal endings optogenetic stimulation and whole-cell voltage-clamp recording of NI neurons. ***B***, Reconstruction of the location of biocytin-filled NI neurons in which IPSCS (blue circles), EPSCS (red circle), or no change (gray circles) in response to optogenetic stimulation of the MS originating axonal endings were recorded. ***C***, Average (±SD) IPSC (20 stimulations) recorded from NI neuron following optogenetic stimulation of MS axonal endings at a command potential of −50 mV and its disappearance at −90 mV. ***D***, Average (±SD) IPSC (20 stimulations) recorded from the NI neuron following optogenetic stimulation of MS axonal endings under control conditions [standard ACSF (sACSF)], its complete disappearance in the presence of GABA_A_ receptor antagonist gabazine (5 μm) and partial return after drug washout. ***E***, Distribution of iPSC amplitudes under control conditions (sACSF), during gabazine application and during washout (*n* = 7). Cyan-colored bars indicate the laser light pulses (465 nm, 5 ms, 10 s interval, <10 mW).

To investigate the functional properties of the MS innervation of the NI, whole-cell voltage-clamp recordings were performed using brain slices from rats that were injected in the MS with AAV2-hSyn-hChR2(H134R)-EYFP (Fig. 12*A*). In 15 of 55 recorded NI neurons, optogenetic stimulation of MS originating fibers evoked IPSCs (Fig. 12*B*,*C*), and only one neuron responded with EPSCs. IPSCs evoked by MS fiber stimulation had a short latency (2.5 ± 0.3 ms, *n* = 15), disappeared at chloride reversal potential (−90 mV; 9 of 9 neurons; Fig. 12*C*), and were blocked by the selective GABA_A_ receptor antagonist gabazine (5 μm; 7 of 7 neurons; Fig. 12*D*,*E*). No differences were detected in the proportions of inhibition/excitation and IPSCs/EPSCs evoked by MS axonal endings stimulation observed in *in vivo* and *ex vivo* recordings, respectively (*p* < 0.05; two-tailed, Fisher's exact test).

### Optogenetic identification of the NI neurons that innervate the MS

**Figure 13.**
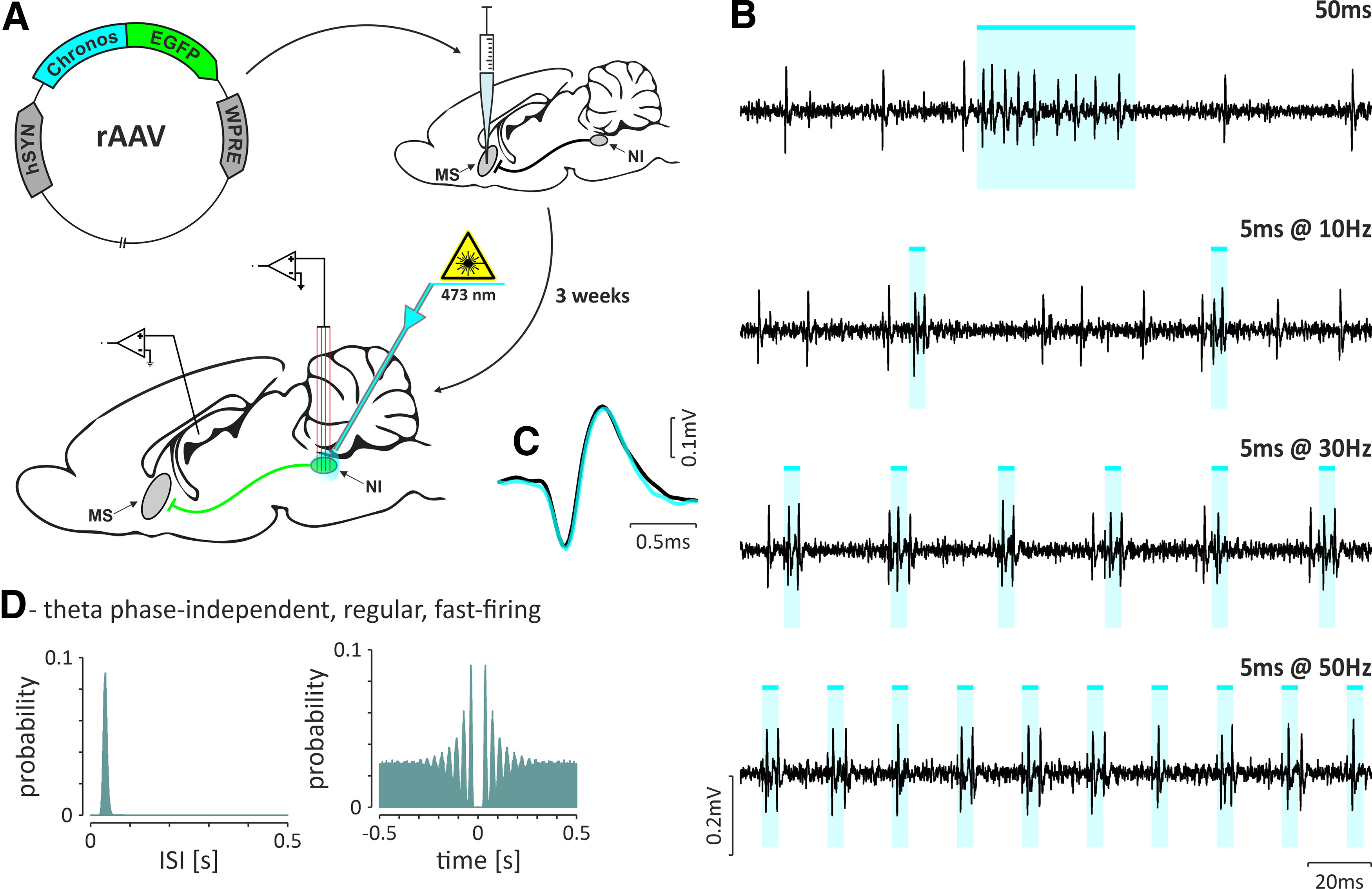
Identification of NI neurons innervating the MS. ***A***, Illustrating the injection of retrograde viral vector into the MS and the experimental procedure conducted 3 weeks after the injection. ***B***, Fragments of raw signal illustrating responses to different optogenetic stimulation protocols: single pulse lasting 50 ms (top) and short, 5 ms pulses at increasing frequencies (10, 30, and 50 Hz). ***C***, Overlaid averaged spike waveforms from the baseline (black) and from periods of light stimulation (cyan). ***D***, Histogram of interspike intervals (left) and autocorrelation function plot (right) of an exemplary theta phase-independent, regular, fast-firing NI neuron that was optogenetically identified as MS projecting (shown in ***B*** and ***C***). Bins, 10 ms. Cyan-colored bars indicate the laser light pulses (473 nm, <10 mW).

In animals with the MS transfected with the retrograde viral vector (pAAV-hSYN-Chronos-GFP; Fig. 13*A*), the direct responses of 22 NI neurons to optogenetic stimulation were observed (Fig. 13*B*; 10 animals; opto-tagged NI neurons/animal: average, 2.2; range, 1–5). All NI neurons identified as innervating MS belonged to the regular, fast-firing theta phase-independent type of cells (100%, 22 of 22; Fig. 13*D*) that, which cortical SWA fired in a delta wave-independent manner (59%, 13 of 22 were irregular, slow firing; 21%, 9 of 22 were regular, slow firing).

## Discussion

In the current study, we identified the following two distinct NI neuronal populations, based on the relationship of their firing to the phase of the hippocampal theta rhythm and on the direction of and interaction with the MS: theta phase-independent and theta phase-locked neurons. Theta phase-independent NI neurons included irregular, slow-firing and regular, fast firing cells. Theta phase-locked neurons had a strong preference to fire action potentials during the rising phase of the hippocampal theta oscillation recorded from the SLM of the hippocampal CA1 field. Notably, theta phase-locked NI neurons could be a bursting or nonbursting type. Theta-bursting NI neurons may have been overlooked in previous studies due to their predominant localisation in the lateral parts of the NI (Ma et al., 2013). During cortical SWA, based on the relation of action potential firing to hippocampal delta waves, we were able to distinguish the following two groups of NI neurons: delta wave independent and delta wave locked. Delta wave-independent NI neurons were of an irregular, slow-firing or regular, fast-firing type, and delta wave-locked neurons were of bursting and nonbursting type. Delta wave-locked NI neurons fired action potentials preferentially on the ascending phase of the delta waves. In line with previous studies, for most NI neurons we noted an increased firing during cortical activation and a decreased discharge rate in the presence of slow-wave activity (Nuñez et al., 2006; Martínez-Bellver et al., 2015). By projection-specific opto-tagging, we determined that only fast-firing theta phase-independent NI neurons innervate the MS. On the other hand, among neurons of this type, a majority were unresponsive to optogenetic stimulation of the MS. In contrast, a majority of theta-bursting NI neurons was inhibited by MS stimulation, and this effect was mediated by direct GABAergic input.

**Figure 14.**
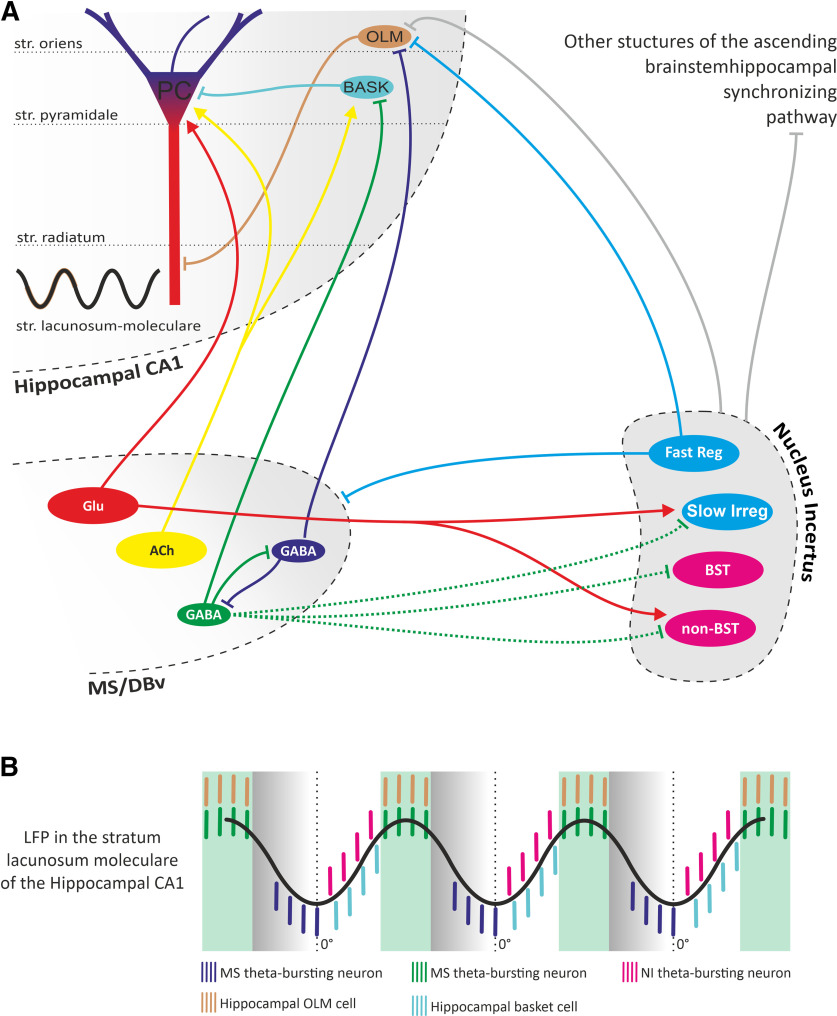
The postulated NI contribution to hippocampal theta rhythm generation. ***A***, A simplified diagram illustrating the nuclei of the septohippocampal system, the NI, and their interconnections. Abbreviations inside the ovals indicate the cell type: ACh, cholinergic; GABA, GABAergic; Glu, glutamatergic within the MS/vertical limb of the diagonal band nucleus (MS/DBv); PC, pyramidal cell; BASK, basket cell; Fast Reg, regular, fast-firing theta phase-independent; Slow Irreg, irregular, slow-firing theta phase-independent; BST, bursting, theta phase-locked; non-BST, non-bursting, theta phase-locked within the NI. Arrowhead endings indicate excitatory connections, and perpendicular endings indicate inhibitory connections. ***B***, Idealized hippocampal theta oscillation observed in the stratum lacunosum-moleculare of the hippocampal CA1 region and schematic temporal distribution of firing of theta-bursting neurons in MS (blue and green bars), NI (purple bars), hippocampal OLM cells (orange bars), and hippocampal basket cell (cyan bars).

Existing knowledge of the NI implies it is a strong modulator of theta oscillations; but the involvement of NI in theta rhythm generation appears to be complex. Fibers originating from the NI heavily innervate the MS, and recent studies showed that GABAergic, including parvalbumin-expressing, and (RXFP3) glutamatergic MS neurons express mRNA for cognate relaxin-3 receptors, which makes these neurons a possible target of a relaxin-3 NI innervation (Olucha-Bordonau et al., 2012; Albert-Gascó et al., 2018; Haidar et al., 2019). Importantly, MS parvalbumin GABAergic neurons were shown to rhythmically hyperpolarize hippocampal interneurons and promote theta oscillations (Hangya et al., 2009). More recent studies in mice revealed that NMB-expressing NI neurons project to the MS and are involved in the control of locomotion, arousal, and hippocampal theta oscillations (Lu et al., 2020; Nasirova et al., 2020). Given that ∼50% of the NMB neurons coexpress RLN3, it is not surprising that the relaxin-3 input from the NI to the MS was also shown to be involved in theta rhythm modulation (Ma et al., 2009; Olucha-Bordonau et al., 2012; Ma et al., 2013; Haidar et al., 2017; Albert-Gascó et al., 2018; Szőnyi et al., 2019). However, until now, the electrophysiological features of the NI neurons innervating the MS were unknown. Our results reveal that only regular, fast-firing theta phase-independent NI neurons project to the MS. In the light of studies by Szőnyi et al. (2019), that revealed that the NI neurons innervating MS send collaterals to oriens lacunosum-moleculare (OLM) neurons of the hippocampus, it can be assumed that the regular, fast-firing NI neurons identified here can also innervate this population of hippocampal interneurons (Fig. 14).

Our data reveal that a majority of the recorded regular, fast-firing theta phase-independent neurons innervating the MS were insensitive to the MS stimulation, and only the minor fractions responded with relatively weak excitation or inhibition. This indicates that the NI neurons innervating the MS do not receive strong MS feedback. At the same time, a majority of theta-bursting neurons responded to the optogenetic stimulation of the MS with initial inhibition followed by a burst of action potentials. This rebound excitation was evident during rhythmic stimulation of the MS with frequencies across the theta band. Additionally, high-frequency stimulation eliminated spontaneous theta bursts, but still synchronized the NI neurons to the frequency of stimulation, which indicates strong control of theta-bursting NI neurons by the input from the MS. Based on these results, it is likely that the NI bursting neurons are innervated by one of the two identified GABAergic medial septal bursting neuron populations, one of which is coupled to the peak and the other to the trough of hippocampal theta wave (observed in the SLM; Borhegyi et al., 2004; Fig. 14).

Theta-bursting MS neurons, by direct innervation of hippocampal interneurons, basket cells, and OLM cells (Fig. 14), produce their rhythmic hyperpolarizations followed by rebound bursts. A similar mechanism may underlie the rhythmic activity of the NI-bursting neurons that receive MS innervation, as described in our study. This is further supported by our observation that higher strength of bursts and intensity of bursting occurred in NI neurons having higher firing rates, while the proportion of action potentials generated in bursts and length of bursts were not correlated with the firing rate. Moreover, the temporal convergence of the bursts of NI neurons and hippocampal basket cells suggests that both these populations may be innervated by the same population of rhythmically bursting GABAergic MS neurons (Fig. 14). In this way, theta-bursting NI neurons can use the pace received from the MS to efficiently participate in the synchronization of oscillatory activity in brain areas involved in locomotion or memory formation (Goto et al., 2001; Olucha-Bordonau et al., 2003). Directional information flows between the NI and the hippocampus, and coherence between the field activity observed in both structures during theta oscillations suggest that the hippocampus is one of the target structures of NI theta-bursting cells (Cervera-Ferri et al., 2011; Martínez-Bellver et al., 2017).

Additionally, our *in vivo* recordings demonstrated that a fraction of the nonbursting theta phase-locked and irregular, slow-firing theta phase-independent NI neurons responded to MS stimulation, and their responses were heterogeneous (inhibition or excitation). At the same time, our *ex vivo* results revealed that MS fiber optostimulation evoked inhibitory, GABA_A_-dependent postsynaptic currents in a vast majority (all but one) of the recorded NI neurons. This suggests that the direct input from MS into NI is largely inhibitory. Concurrently, a recent study in mice has shown that the stimulation of MS terminals within the NI elicited both GABA- and glutamate-dependent responses (Lu et al., 2020). These results indicate possible species differences in MS-NI innervation, as shown for some features of the NI originating RLN3/RXFP3 system in mice and rats (Ma et al., 2017a).

Several studies have reported that NI activation or inhibition affects theta oscillations (Nuñez et al., 2006; Martínez-Bellver et al., 2017; Ma et al., 2017b; Szőnyi et al., 2019; Lu et al., 2020), but the resultant interpretation remains inconclusive. Some of the discrepancies between studies may result from methodological differences, such as the species used (mouse/rat), the state of the animal (anesthetized/freely moving), the stimulation method (electrical/optogenetic), or the group of activated cells (GABAergic/RLN3-positive/NMB-positive). In one recent study, all GABAergic NI neurons were optogenetically activated with light pulses applied at 25 Hz (Szőnyi et al., 2019), which resulted in a decrease in the power of hippocampal theta oscillations. Such a stimulation protocol—while inducing activity of regular, fast-firing theta phase-independent NI neurons that is close to their physiological rate and pattern of firing—at the same time can desynchronize rhythmic firing of the theta-bursting NI neurons. If, as we postulate from our observations, the function of theta-bursting NI neurons is to enhance the synchronization in theta frequencies, the tonic activity of these neurons, forced by optogenetic stimulation, should weaken the theta oscillation strength. This explanation is further supported by the results reported by Lu et al. (2020), who increased the power of hippocampal theta oscillations by selectively stimulating the NI neuronal population innervating the MS (and therefore not theta-bursting NI neurons, as we know from our observations), with light pulses at a frequency and a pattern similar to their physiological firing (our study; Lu et al., 2020). The current and previous data (Nuñez et al., 2006; Lu et al., 2020) indicate that the innervation of the MS by regularly (tonically) firing NI neurons has a facilitating and/or gating function in the brainstem originating mechanism of theta rhythm induction. In line with these results, Ma et al. (2017b), using activation of the NI via hM3Dq-DREADD (designer receptors exclusively activated by designer drugs), demonstrated that NI neuron depolarization—plausibly maintaining their firing pattern—caused an increase in theta power. In the case of the theta-bursting population of neurons, this probably led to the generation of more action potentials in bursts and possible transition of irregularly bursting neurons into the rhythmic theta-bursting firing mode.

Hippocampal theta oscillations result from rhythmic changes in polarization of the principal cells, induced by the activity of hippocampal interneurons, which in turn are influenced by signals from the MS. In our proposed model, two distinct neuronal populations, identified in the NI, may be involved in hippocampal theta rhythm generation in different ways: regular, fast-firing theta phase-independent NI neurons innervating the MS may have permissive function, while theta-bursting NI neurons are likely involved in synchronizing theta rhythm of other brain structures (Fig. 14). In summary, our results shed new light on the interpretation of previous observations and provide the basis for a more precise incorporation of the NI in the mechanism of generation and/or modulation of septohippocampal theta oscillations.
